# Effects of exercise interventions based on American College of Sports Medicine recommendations on physical function, body composition, and quality of life in older adults with sarcopenia: a meta-analysis of randomized controlled trials

**DOI:** 10.3389/fphys.2026.1882998

**Published:** 2026-10-09

**Authors:** Yanan Zhang, Xiaonan Liu, Chun Tian, Shuai Tao, Huan Wang

**Affiliations:** Beijing Tiantan Hospital Affiliated to the Capital University of Medical Sciences, Nursing Operating Room, Beijing, China

**Keywords:** American College of Sports Medicine, body composition, exercise, meta-analysis, sarcopenia in older adults, nursing

## Abstract

**Abstract:**

Aging is associated with a progressive decline in skeletal muscle mass and strength, which adversely affects the quality of life in older adults. This study aimed to investigate the effects of different exercise volumes on physical function, body composition, and quality of life in older adults with sarcopenia.

**Methods:**

Randomized controlled trials (RCTs) published up to February 28,2026 that investigated the effects of exercise interventions on older adults with sarcopenia were systematically retrieved from PubMed, Embase, Web of Science, and the Cochrane Library. Study quality was assessed using the Cochrane Risk of Bias tool, and meta-analyses were conducted using appropriate statistical software. Subgroup analyses were performed to examine the influence of adherence to the American College of Sports Medicine (ACSM) guidelines on outcome measures. In addition, sensitivity analyses were conducted to evaluate the robustness of the pooled results, while publication bias was assessed using funnel plots and Egger’s regression test to ensure the comprehensiveness and reliability of the findings.

**Results:**

The meta-analysis demonstrated that, compared with the control groups, exercise interventions significantly improved handgrip strength (standardized mean difference (SMD) = 0.47, 95% confidence interval (CI): 0.28, 0.66, P < 0.01), gait speed (SMD = 0.70, 95% CI: 0.45, 0.96, P < 0.01), walking test performance (SMD = −0.73, 95% CI: −1.14, −0.32, P < 0.01), skeletal muscle index (SMD = 0.48, 95% CI: 0.19, 0.77, P < 0.01) and body fat percentage (SMD = −0.62, 95% CI: −1.07, −0.17, P < 0.01). Subgroup analyses indicated that improvements in handgrip strength, gait speed, walking test performance, skeletal muscle index, bone mineral density, and health status and quality of life were significantly greater in the high-adherence group than in the low-adherence group (P <0.05).Overall, appropriately designed exercise programs can significantly enhance physical function and body composition, whereas improvements in health status and quality of life depend on high adherence to ACSM recommendations.

**Conclusion:**

Compared with patients with low adherence, older adults with sarcopenia who demonstrated high adherence to exercise interventions experienced more pronounced improvements in physical function, body composition, and quality of life. However, these findings should be interpreted with caution and require confirmation through large-scale RCTs with longer follow-up durations.

**Systematic Review Registration:**

https://www.crd.york.ac.uk, identifier CRD420261297264.

## 1 Introduction

Introduction (Chen et al., 2020). Sarcopenia is associated with a wide range of adverse outcomes, including falls and secondary fractures (Harris et al., 2017; Yeung et al., 2019), impaired pulmonary function (Ohara et al., 2020), sleep disturbances (Ida et al., 2019), cognitive dysfunction (Peng et al., 2020), reduced health-related quality of life (Rizzoli et al., 2013), and premature mortality (Kitamura et al., 2021), all of which impose substantial medical and economic burdens. In addition, sarcopenia significantly increases the risk of hospitalization among older adults (Zhang et al., 2018; Yang et al., 2019). Compared with older individuals without sarcopenia, those with preoperative sarcopenia incur nearly USD 13,000 higher total hospitalization costs (Gani et al., 2016). In 2000, healthcare expenditures related to sarcopenia-associated conditions in the United States reached USD 18.5 billion, accounting for approximately 1.5% of total annual healthcare spending (Janssen et al., 2004). Therefore, the prevention and management of sarcopenia are critical for preserving physical function, improving health outcomes, and reducing healthcare-related costs in the aging population.

The World Health Organization (WHO) 2020 guidelines recommend that older adults engage in at least 150 minutes of moderate-intensity or 75 minutes of vigorous-intensity physical activity per week, in addition to performing resistance training at least twice weekly; increasing daily step counts has also been shown to reduce the risk of sarcopenia. Moreover, sarcopenia is directly associated with insufficient physical activity (Billot et al., 2020; Ko et al., 2021). A growing body of evidence indicates that increased physical activity can effectively enhance muscle strength and muscle mass in older adults (Kosek et al., 2006; Candow et al., 2011). Furthermore, a systematic review and meta-analysis including 25 studies demonstrated that physical activity significantly reduces the prevalence of sarcopenia among older populations (Steffl et al., 2017).

Meta-analyses synthesize findings from multiple primary studies to identify optimal exercise doses, thereby strengthening the overall level of evidence. However, heterogeneity arising from differences in diagnostic criteria, participant characteristics, and methodological approaches poses significant challenges to data consistency in meta-analyses, potentially reducing statistical power (Chen et al., 2021). These limitations are particularly evident in evaluations of the dose–response effects of resistance training (RT) among older adults with sarcopenia (Chen et al., 2021; Talar et al., 2021; Zhang et al., 2021; Mende et al., 2022). Moreover, existing analyses often lack a comprehensive and detailed examination of training variables and other influential factors, such as muscle contraction velocity and sample size considerations. Notably, to date, no meta-analysis has systematically incorporated exercise guidelines established by the ACSM when examining the effects of exercise interventions on outcome measures in older adults with sarcopenia.

Therefore, this study aimed to identify the optimal exercise volume for the treatment and prevention of sarcopenia in older adults. *T*he ACSM provides evidence-based recommendations for exercise prescription based on the FITT principle (frequency, intensity, time, and type), which have been applied across a range of clinical populations (Riebe et al., 2015; Parke et al., 2022). To quantify adherence to these recommendations in older adults with sarcopenia, we classified each included intervention according to whether its frequency, intensity, time, and type met the ACSM-recommended thresholds, thereby enabling a dose–response comparison (high vs. low adherence). However, it remains unclear whether exercise interventions based on ACSM guidelines confer greater benefits for older adults with sarcopenia. Therefore, the objective of this study was to compare the effects of high versus low adherence to ACSM guidelines on physical function, body composition, and quality of life in older adults with sarcopenia.

## 2 Materials and methods

This systematic review was conducted according to the guidelines of the Cochrane Collaboration and the Preferred Reporting Items for Systematic Reviews and Meta-Analyses (PRISMA) statement (Page et al., 2021) and was prospectively registered with PROSPERO (CRD420261297264) prior to the initiation of study screening and data extraction.

### 2.1 Search strategy

To ensure the robustness of the evidence, a comprehensive and systematic search was conducted using controlled vocabulary from the Medical Subject Headings (MeSH) database in combination with relevant keywords to identify all eligible RCTs published from database inception to February 28,2026. The databases searched included PubMed, Embase, Web of Science and the Cochrane Library. The search strategy was developed in accordance with the PICOS framework, with particular emphasis on the study population, interventions, and study design. Searches were performed using combinations of MeSH terms and free-text keywords. Detailed search strategies are provided in **Supplementary Material 1**. In addition, reference lists of relevant review articles were manually screened, and additional eligible studies were identified through supplementary searches. When necessary, the corresponding authors were contacted to obtain further information as presented in **Table 1**. (PubMed is used as an example).

**Table 1:** Search strategy.

#1	sarcopenia[MeSH Terms]
#2	(sarcopenia[Title/Abstract]) OR (sarcopenias[Title/Abstract])
#3	(#1) AND (#2)
#4	(((((((exercise[MeSH Terms]) OR (Resistance Training[MeSH Terms])) OR (Plyometric Exercise[MeSH Terms])) OR (Exercise Movement Techniques[MeSH Terms])) OR (Exercise Therapy[MeSH Terms])) OR (Endurance Training[MeSH Terms])) OR (Tai Ji[MeSH Terms])) OR (Yoga[MeSH Terms])
#5	((((((((((((((((((((((((Exercise[Title/Abstract]) OR (Exercises[Title/Abstract])) OR (Resistance Training[Title/Abstract])) OR (Plyometric Exercise[Title/Abstract])) OR (Exercise Movement Techniques[Title/Abstract])) OR (Endurance Training[Title/Abstract])) OR (Exercise Therapy[Title/Abstract])) OR (Tai Ji[Title/Abstract])) OR (Yoga[Title/Abstract])) OR (Qigong[Title/Abstract])) OR (Baduanjin[Title/Abstract])) OR (Yijinjing[Title/Abstract])) OR (Exercise, Physical[Title/Abstract])) OR (Exercises, Physical[Title/Abstract])) OR (Physical Exercise[Title/Abstract])) OR (Physical Exercises[Title/Abstract])) OR (Exercise, Aerobic[Title/Abstract])) OR (Aerobic Exercise[Title/Abstract])) OR (Aerobic Exercises[Title/Abstract])) OR (Exercises, Aerobic[Title/Abstract])) OR (Exercise, Isometric[Title/Abstract])) OR (Exercises, Isometric[Title/Abstract])) OR (Isometric Exercises[Title/Abstract])) OR (Isometric Exercise[Title/Abstract])) OR (Acute Exercise[Title/Abstract])
#6	(#4) AND (#5)
#7	(#3) AND (#6)

### 2.2 Inclusion and exclusion criteria

The criteria for inclusion and exclusion were determined according to the PICOS framework (Participants, Interventions, Comparators, Outcomes, and Study design) (Schardt et al., 2007), as presented in **Table 2**. Using these predefined standards, two reviewers independently assessed potentially eligible articles at the title, abstract, and full-text levels to confirm their suitability.

**Table 2:** PICOS framework.

Items	Inclusion criteria	Exclusion criteria
Population (P)	The study population was diagnosed with sarcopenia	The study population was not diagnosed with sarcopenia at baseline
Intervention (I)	exercise interventions in the experimental group, including any form of physical active exercise such as cardiorespiratory exercise, resistance training, or flexibility training	exercise interventions in the experimental group, including any form of physical passive exercise such as whole body electrical stimulation therapy;Whole body vibration
Comparator (C)	conventional treatment in the control group, such as routine care or pharmacological therapy, with no exercise intervention	Studies with no control group (e.g., single-arm trials)
Outcome (O)	Outcome measures including handgrip strength, skeletal muscle index, and gait speed,Walking Test,Chair Stand Test,and so on.	studies with incomplete or unreported data
Study Design (S)	Published randomized studies were included in the study design	Non-randomized studies (e.g., cohort studies, case-control studies, observational studies)

### 2.3 Data synthesis and analysis

Data extraction was independently performed by two authors (YN and TC). The primary outcome measures of interest in this study included handgrip strength, skeletal muscle index, and gait speed. A standardized Excel spreadsheet was predesigned to extract relevant data, including publication characteristics (title, authors, year of publication, and country), methodological characteristics (number of study arms, intervention protocols, and sample size), participant characteristics (age and sex ratio), exercise characteristics (intervention frequency, exercise intensity, duration, number of repetitions, and sets), as well as risk of bias assessments and outcome data. When outcome data were presented graphically without explicit numerical values, Engauge Digitizer software was used to extract the required data. For studies with multiple follow-up time points, only data obtained immediately after the intervention were extracted.

### 2.4 ACSM evaluation criteria

After data extraction, the exercise intervention dose and adherence were systematically evaluated. The exercise prescriptions of the included studies were assessed according to the recommendations outlined in the ACSM guidelines for the development and maintenance of cardiorespiratory and muscular fitness in patients with cancer (Page et al., 2021). Two authors (TS and WH) independently evaluated and scored each component of the exercise interventions, including frequency, intensity, and duration, based on the ACSM-recommended criteria to assess exercise adherence and effectiveness as presented in **Table 3**.

**Table 3:** ACSM recommendations for cardiorespiratory fitness, muscular strength, and flexibility in apparently healthy adults.

Exercise dose	Cardiorespiratory exercise	Resistance exercise	Flexibility exercise
Frequency	3 days per week	2–3 days per week	More effective on ≥ 5 days per week, daily
Intensity/workload	40–60% VO2R or HRR; RPE of 12–13 on a 6–20 scale	Start with 40–50% 1RM, more capable with 60–70% 1RM	Full range of flexion, extension and rotation
Duration	Continuous or cumulative 30 min	≥1 group, 8–12 repetitions; patients started with 10–15 repetitions	Keep static pulling for 10–30s; repeat 2–4 times

ACSM, American College of Sports Medicine; HRR, heart rate reserve; VO2R, oxygen uptake reserve; RPE, rating of perceived exertion; 1RM one repetition maximum.

cardiorespiratory exercise was defined as follows: moderate intensity corresponded to 40–59% of maximal oxygen uptake (VO_2_max) or heart rate reserve (HRR), whereas high intensity was defined as ≥60% of VO_2_max or HRR, with individualization as required. Resistance exercise was categorized as moderate intensity when the final two sets were performed with a moderate level of challenge (e.g., the final set could be completed but with noticeable difficulty), and as high intensity when the training load was increased, provided that good tolerance and safety were confirmed. Flexibility exercise involved stretching the targeted muscles to a point of tension or mild discomfort, holding the stretch for 10–30 seconds, and repeating each stretch 2–4 times.

In the scoring system, a score of 1 indicated a strong recommendation, whereas a score of 0 indicated no recommendation. Exercise adherence scores ranged from 0 to 2, with a score of 2 indicating compliance with the criteria, a score of 1 indicating uncertainty, and a score of 0 indicating non-compliance with the criteria.

When discrepancies arose between the two reviewers, consensus was reached through discussion with a third author. Based on this scoring framework, the proportion of exercise dose adherence in each study was calculated to determine compliance with the ACSM recommended doses.

### 2.5 Data analysis

Meta-analyses were performed using Review Manager (RevMan) version 5.4 to synthesize and compare outcomes across the included studies. Studies were categorized into two groups according to their level of adherence to ACSM recommendations, defined as high adherence or low/uncertain adherence. Given the variability in exercise frequency and duration across the included trials, a random-effects model was applied. Heterogeneity within each subgroup was assessed using the Higgins I statistic, which quantifies the proportion of total variation across studies attributable to heterogeneity rather than chance. Expressed as a percentage, a higher I value indicates greater inconsistency among study results and may necessitate further investigation into potential sources of heterogeneity. In accordance with the Cochrane Handbook guidelines (Deeks, 2011). heterogeneity was categorized as low (≤ 25%), moderate (25% < I ≤ 50%), substantial (50% < I ≤ 75%), or considerable (I > 75%). Effect sizes were expressed as standardized mean differences (SMDs) with corresponding 95% confidence intervals (95% CI). Potential publication bias was evaluated by visual inspection of funnel plots constructed from study effect sizes and their standard errors and further assessed using Begg’s rank correlation test and Egger’s linear regression test, with P < 0.05 considered statistically significant.

### 2.6 Quality assessment

Study quality was independently assessed by two reviewers (YN and TS) in accordance with the Cochrane-recommended criteria for evaluating randomized controlled trials (Higgins et al., 2011), using the methodological framework of the Cochrane Collaboration to assess the risk of bias. The following domains were evaluated: random sequence generation (selection bias), allocation concealment (performance bias), blinding of participants and personnel (performance bias), blinding of outcome assessment (detection bias), incomplete outcome data (attrition bias), selective reporting (reporting bias), and other potential sources of bias, as recommended (Sterne et al., 2019). When additional information was required, the corresponding authors were contacted, and any discrepancies between reviewers were resolved through discussion until consensus was reached.

## 3 Results

### 3.1 Study selection

A total of 4,108 records were identified through database searches, including PubMed (n = 988), the Cochrane Library (n = 1,808), Embase (n = 595), and Web of Science (n = 717). After removing 504 duplicate records, 3,604 articles remained for further screening. Following the review of titles and abstracts, 728 studies were identified as potentially meeting the inclusion criteria. After full-text assessment, 33 studies were ultimately included in the meta-analysis (Gadelha et al., 2016; Hassan et al., 2016; Maruya et al., 2016; Vasconcelos et al., 2016; Hamaguchi et al., 2017; Liao et al., 2017; Chen et al., 2018; Liao et al., 2018; Najafi et al., 2018; Jung et al., 2019; Lichtenberg et al., 2019; Vikberg et al., 2019; Kemmler et al., 2020b; Kemmler et al., 2020a; Makizako et al., 2020; Oh et al., 2020; Kemmler et al., 2021; Sen et al., 2021; Seo et al., 2021; Chien et al., 2022; Jung et al., 2022; Lin et al., 2022; Yuenyongchaiwat and Akekawatchai, 2022; Chen et al., 2023; Flor-Rufino et al., 2023a; Flor-Rufino et al., 2023b; Monti et al., 2023; Nie et al., 2023; Xiao et al., 2023; Chen and Zhang, 2024; Liu et al., 2024; Rezaei et al., 2024; Wu et al., 2024) are shown in **Figure 1**.

**FIGURE 1:**
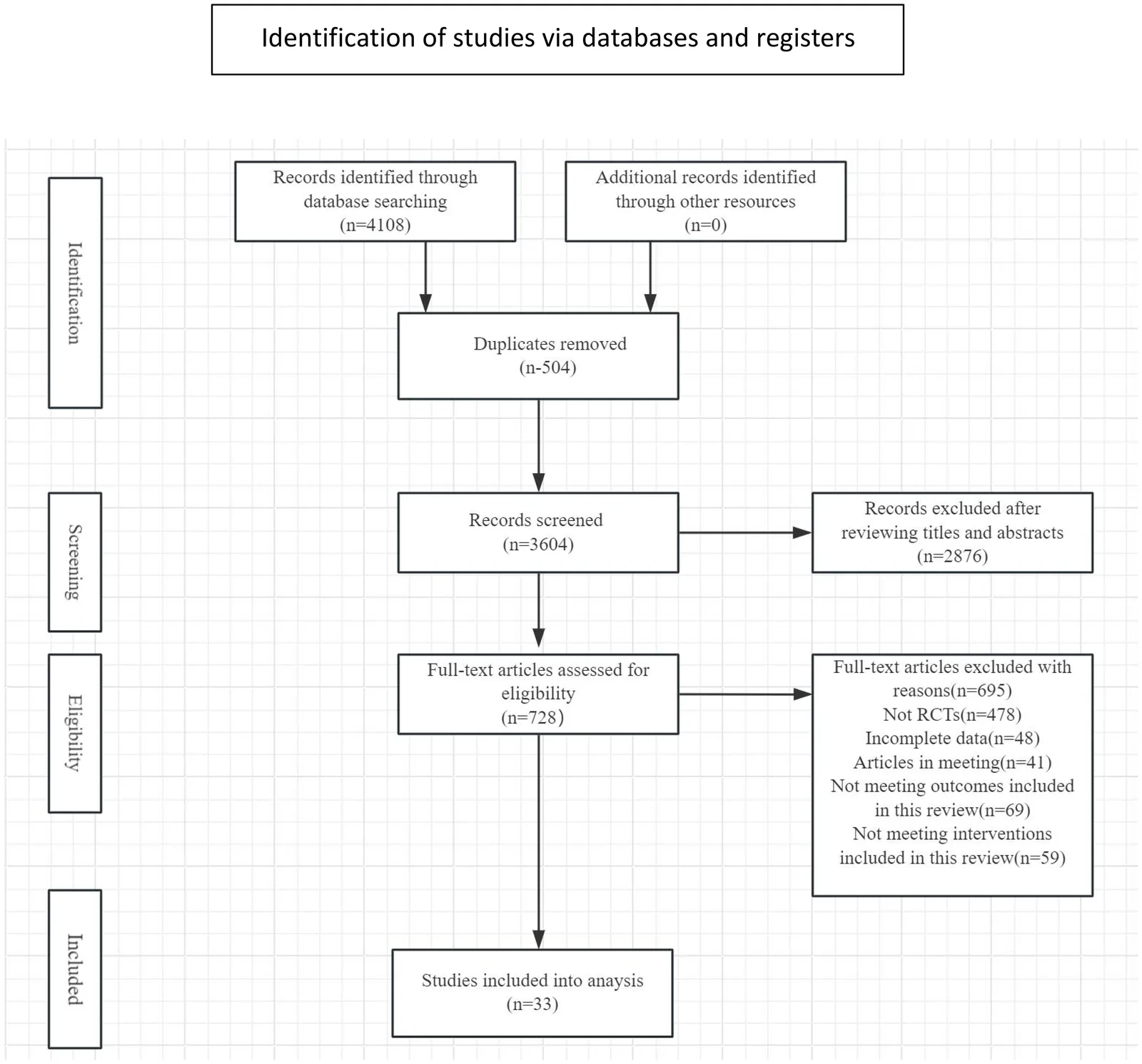
PRISMA study flow diagram.

### 3.2 Study characteristics

The 33 included studies enrolled a total of 2,217 participants, with 1,134 assigned to the intervention groups and 1,083 to the control groups. In terms of geographical distribution, two studies were conducted in Spain, two in Brazil, three in Korea, nine in China, two in Iran, four in Taiwan, three in Germany, and three in Japan, while one study each was carried out in Thailand, Italy, Sweden, Turkey, Australia, and Hong Kong. Information on publication year, participant age in both groups, intervention modalities, and outcome measures was also extracted. Of the 33 studies, 11 included cardiorespiratory exercise, 29 incorporated resistance training, and 5 included flexibility training (some studies included more than one modality).According to the recommendations of the ACSM, all 33 studies were compliant with ACSM guidelines as presented in **Supplementary Files 1**, **2**.

### 3.3 Risk of bias

The risk-of-bias assessment showed that 33 studies were included, and none were judged to have an overall low risk of bias. Specifically, 18 studies (54.5%) were rated as having a high risk of bias, while 15 studies (45.5%) were judged to have some concerns. Regarding specific domains, 16 studies were assessed as low risk, 15 as having some concerns, and 2 as high risk in the randomization process; 28 studies had some concerns and 5 were rated as high risk due to deviations from the intended interventions; 12 studies were rated as low risk, 15 as having some concerns, and 6 as high risk due to missing outcome data; 19 studies were assessed as low risk, 13 as having some concerns, and 1 as high risk in outcome measurement; and 19 studies had some concerns while 14 were rated as high risk in the selection of the reported result. Overall, the main sources of bias were deviations from the intended interventions, missing outcome data, and selective reporting, indicating that the findings should be interpreted with caution. Are shown in **Figure 2**.

**Figure 2:**
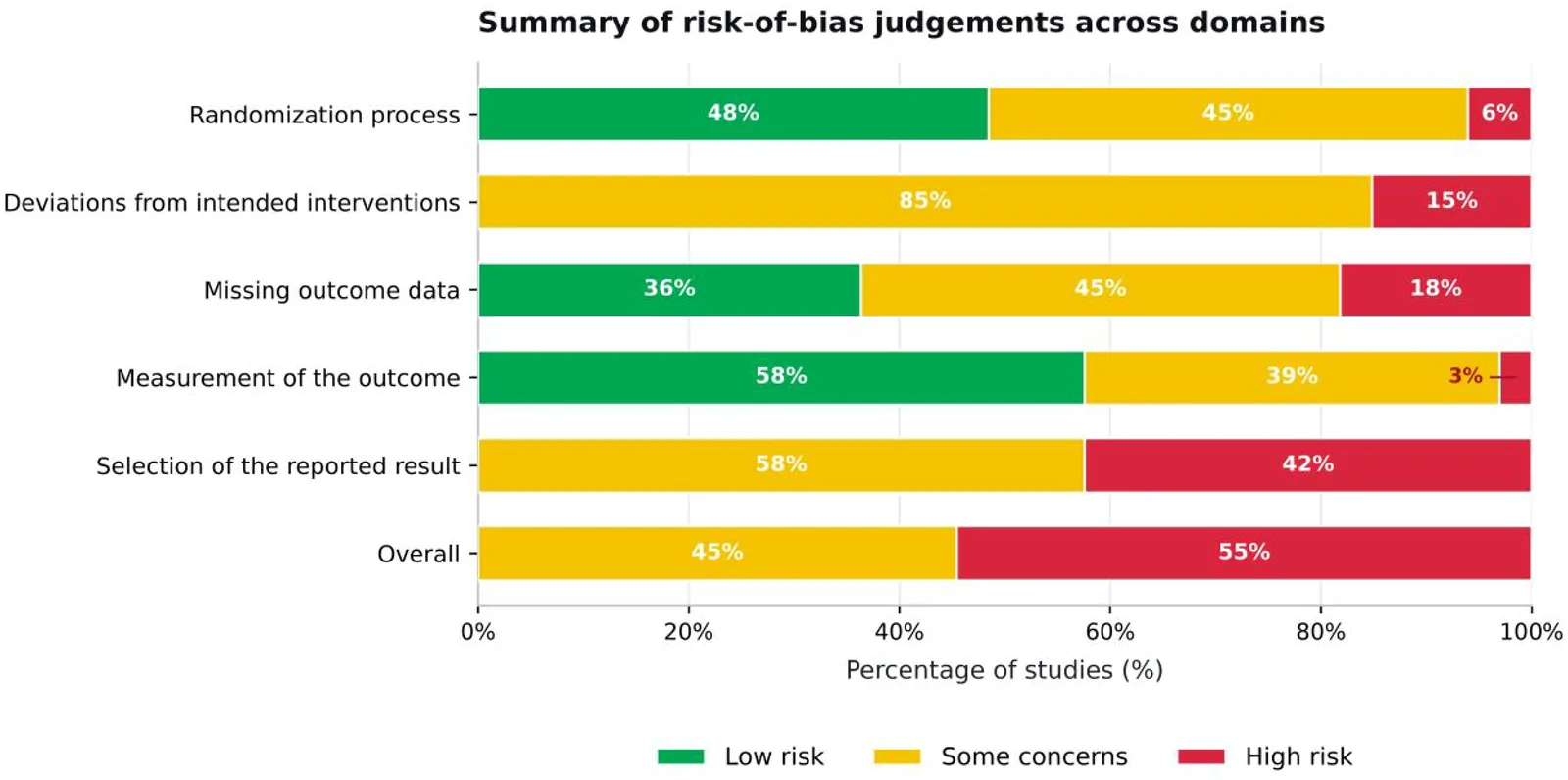
Risk of bias summary: The review author’s judgement of the risk of bias of each included study.

### 3.4 Outcome measures

#### 3.4.1 Physical function

##### 3.4.1.1 Handgrip strength

Heterogeneity was first assessed, yielding an I²value of 62% with P<0.001, indicating moderate heterogeneity; therefore, a random-effects model was applied for the statistical analysis. The pooled results showed an overall SMD = 0.47 (95% CI: 0.28, 0.66, P<0.001), suggesting that exercise interventions significantly improved handgrip strength. In the subgroup analysis, studies were stratified according to the level of adherence to the ACSM recommendations. The subgroup with high ACSM adherence demonstrated a pooled SMD = 0.62 (95% CI: 0.40, 0.85, P<0.001), whereas the subgroup with low or unclear ACSM adherence showed a pooled SMD = 0.16 (95% CI: −0.05, 0.37, P = 0.13). The test for subgroup differences indicated a statistically significant difference between exercise interventions with high ACSM adherence and those with low adherence (P = 0.003). (Forest plot, funnel plot, subgroup Forest plot see **Figures 3**–**5**). These findings suggest that, among older adults with sarcopenia, exercise interventions adhering closely to ACSM recommendations are more effective in improving handgrip strength than those with lower or uncertain adherence.

**Figure 3:**
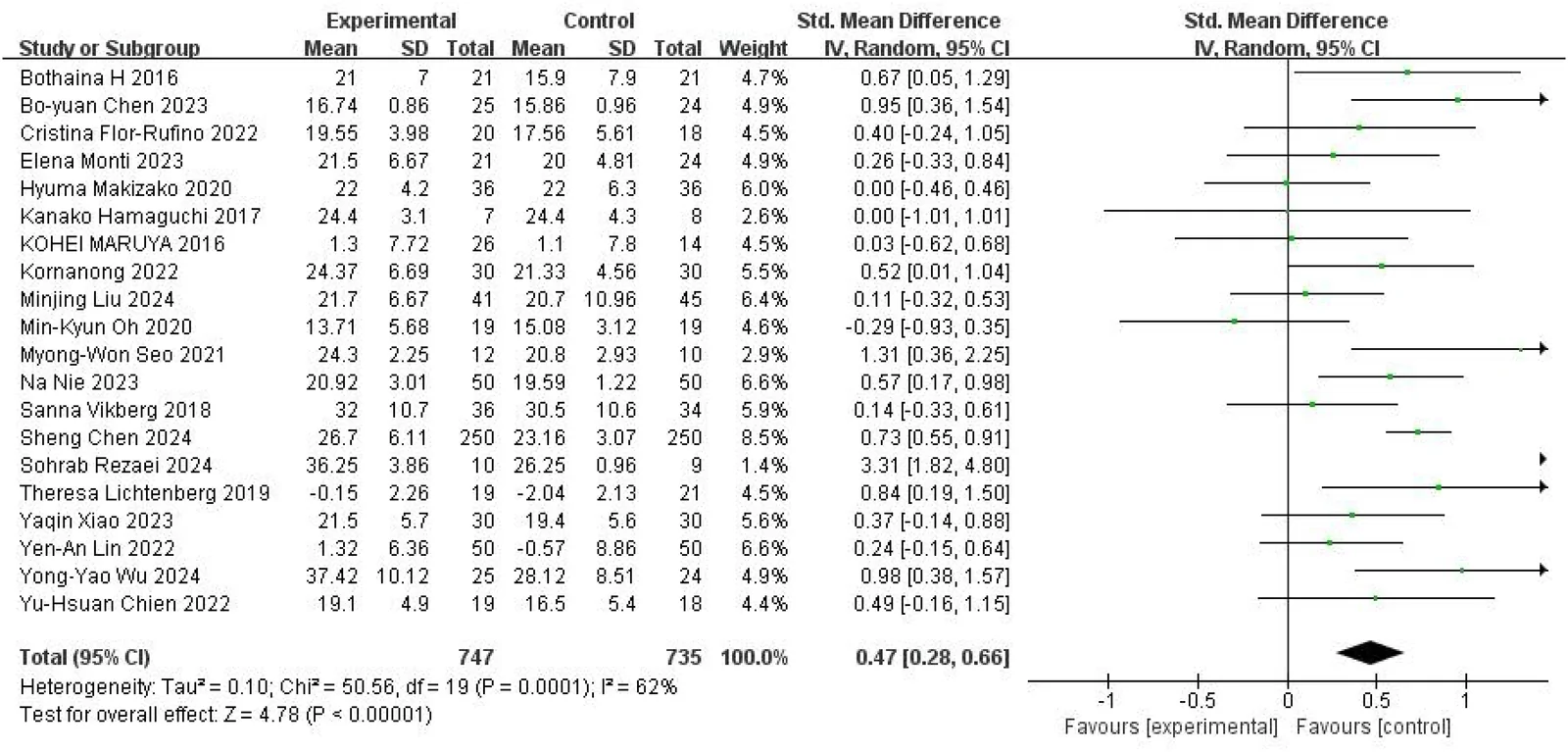
Forest plot of the pooled effect of exercise on handgrip strength across twenty randomized controlled trials. Heterogeneity: I² = 62%, P < 0.001. A random-effects model was used. The pooled standardized mean difference was 0.47 (95% CI: 0.28, 0.66; P < 0.01), indicating a significant improvement in handgrip strength favoring the exercise group.

**Figure 4:**
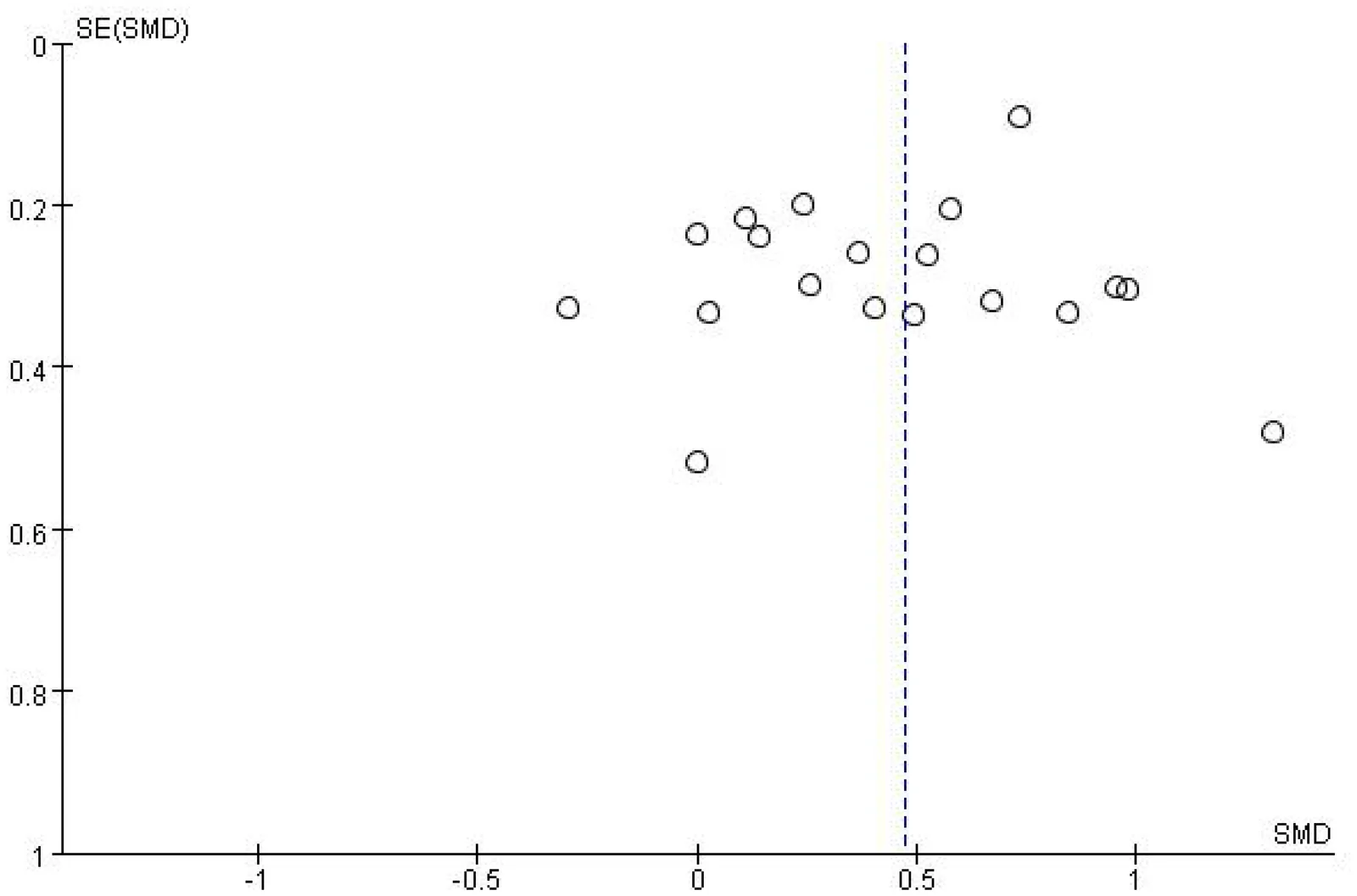
Funnel plot of the pooled effect of exercise on handgrip strength across twenty randomized controlled trials. Heterogeneity: I² = 62%, P < 0.001. A random-effects model was used. The pooled standardized mean difference was 0.47 (95% CI: 0.28, 0.66; P < 0.01), indicating a significant improvement in handgrip strength favoring the exercise group.

**Figure 5:**
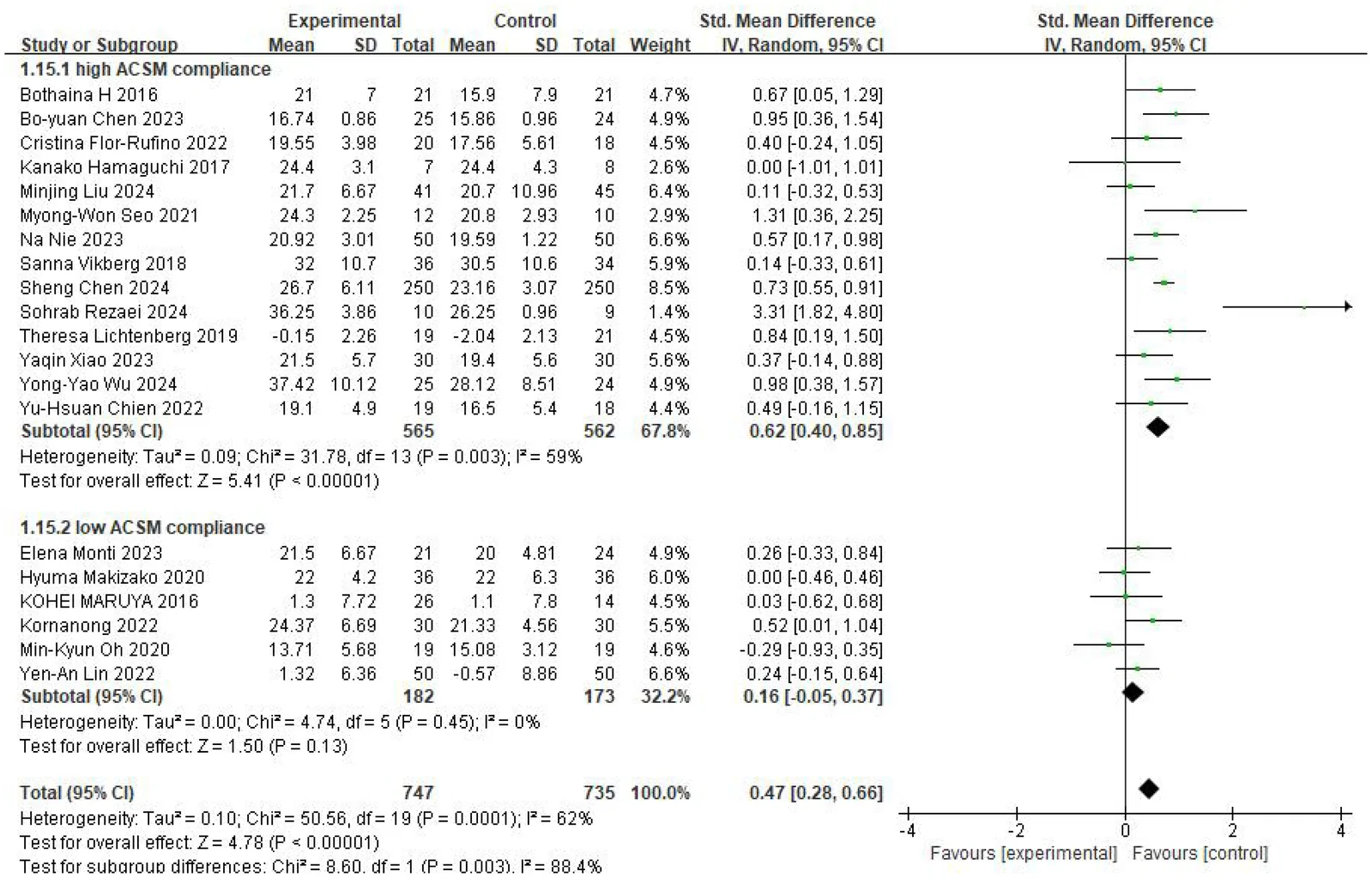
Forest plot of the effects of high compliance and low compliance on grip strength across twenty randomized controlled trials. Heterogeneity: I² = 62%, P < 0.001. A random-effects model was used. The pooled standardized mean difference was 0.47 (95% CI: 0.28, 0.66; P = 0.003), indicating a significant improvement in handgrip strength favoring the exercise group.

##### 3.4.1.2 Gait speed

Heterogeneity was initially assessed and revealed an I² value of 76% with P<0.001, indicating moderate heterogeneity; therefore, a random-effects model was applied. The pooled analysis showed an overall SMD = 0.70 (95% CI: 0.45, 0.96, P<0.001), indicating that exercise interventions significantly improved gait speed. In the subgroup analysis, studies were categorized according to the level of adherence to the ACSM recommendations. The subgroup with high ACSM adherence demonstrated a pooled SMD = 0.90 (95% CI: 0.57, 1.24, P<0.001), whereas the subgroup with low or unclear ACSM adherence showed a pooled SMD = 0.30 (95% CI: −0.02, 0.61, P = 0.06). The test for subgroup differences indicated a statistically significant difference between exercise interventions with high and low ACSM adherence (P = 0.01) (Funnel plot, Forest plot, subgroup Forest plot see **Supplementary Figure 1**). These results suggest that, among older adults with sarcopenia, exercise interventions with high adherence to ACSM recommendations are more effective in improving gait speed than those with lower or uncertain adherence.

##### 3.4.1.3 Walking test

Heterogeneity was first evaluated and yielded an I² value of 79% with P<0.001, indicating moderate heterogeneity; therefore, a random-effects model was used for the analysis. The pooled results demonstrated an overall SMD =−0.73 (95% CI: −1.14, −0.32, P<0.001), indicating that exercise interventions significantly improved walking test performance. In the subgroup analysis, studies were stratified according to the level of adherence to the ACSM recommendations. The subgroup with high ACSM adherence showed a pooled SMD = −0.82 (95% CI: −1.26,−0.39, P<0.001), whereas the subgroup with low or unclear ACSM adherence yielded a pooled SMD = −0.02 (95% CI: −0.52, 0.49, P<0.001). The test for subgroup differences revealed a statistically significant difference between exercise interventions with high and low ACSM adherence (P = 0.02) (Funnel plot, Forest plot, subgroup Forest plot see **Supplementary Figure 2**). These findings indicate that, among older adults with sarcopenia, exercise interventions with high adherence to ACSM recommendations are substantially more effective in improving walking test outcomes than those with lower or uncertain adherence.

#### 3.4.2 Body composition

##### 3.4.2.1 Skeletal muscle index

Heterogeneity testing indicated a high level of heterogeneity (I²=80%, P<0.001); therefore, a random-effects model was employed. The pooled analysis demonstrated an overall SMD = 0.48 (95% CI: 0.19, 0.77, P = 0.001), suggesting that exercise interventions significantly improved the skeletal muscle index. In the subgroup analysis, studies were stratified according to adherence to the ACSM recommendations. The subgroup with high ACSM adherence showed a pooled SMD = 0.62 (95% CI: 0.27, 0.96, P = 0.004), whereas the subgroup with low or unclear ACSM adherence yielded a pooled SMD = −0.04 (95% CI: −0.36, 0.28, P = 0.82). The test for subgroup differences revealed a statistically significant difference between exercise interventions with high and low ACSM adherence (P = 0.006) (Funnel plot, Forest plot, subgroup Forest plot see **Supplementary Figure 3**). These findings indicate that, among older adults with sarcopenia, exercise interventions with high adherence to ACSM recommendations are more effective in improving the skeletal muscle index than those with lower or uncertain adherence.

##### 3.4.2.2 Body mass

Heterogeneity testing showed substantial heterogeneity (I²=60%, P = 0.01); therefore, a random-effects model was applied. The pooled analysis yielded an overall SMD =−0.13 (95% CI: −0.51, 0.24, P = 0.48), indicating that exercise interventions did not result in a significant improvement in Body Mass. In the subgroup analysis, studies were stratified according to adherence to the ACSM recommendations. The subgroup with high ACSM adherence demonstrated a pooled SMD = −0.08 (95% CI: −0.50, 0.34, P = 0.71), whereas the subgroup with low or unclear ACSM adherence showed a pooled SMD = −0.49 (95% CI: −1.24, 0.27, P = 0.21). The test for subgroup differences indicated no statistically significant difference between exercise interventions with high and low ACSM adherence (P = 0.35) (Funnel plot, Forest plot, subgroup Forest plot see **Supplementary Figure 4**). These results suggest that, among older adults with sarcopenia, higher adherence to ACSM recommendations does not confer a clear advantage over lower or uncertain adherence in improving Body Mass outcomes.

##### 3.4.2.3 Body mass index

Heterogeneity testing revealed substantial heterogeneity (I²=65%, P = 0.001); therefore, a random-effects model was applied. The pooled analysis showed an overall SMD = 0.04 (95% CI: −0.26, 0.34, P = 0.79), indicating that exercise interventions did not significantly improve body mass index. In the subgroup analysis, studies were stratified according to adherence to the ACSM recommendations. The subgroup with high ACSM adherence demonstrated a pooled SMD = −0.01 (95% CI: −0.41, 0.39, P = 0.96), whereas the subgroup with low or unclear ACSM adherence yielded a pooled SMD = 0.12 (95% CI: −0.24, 0.47, P = 0.52). The test for subgroup differences indicated no statistically significant difference between exercise interventions with high and low ACSM adherence (P = 0.64)(Funnel plot, Forest plot, subgroup Forest plot see **Supplementary Figure 5**). These findings suggest that, among older adults with sarcopenia, higher adherence to ACSM recommendations does not provide a clear advantage over lower or uncertain adherence in improving body mass index outcomes.

##### 3.4.2.4 Lean body mass

Heterogeneity testing indicated moderate heterogeneity (I²=50%, P = 0.06); therefore, a random-effects model was applied. The pooled analysis yielded an overall SMD = 0.23 (95% CI: −0.08, 0.53, P = 0.15), suggesting that exercise interventions did not result in a statistically significant improvement in lean body mass. In the subgroup analysis, studies were stratified according to adherence to the ACSM recommendations. The subgroup with high ACSM adherence showed a pooled SMD = 0.32 (95% CI: −0.01, 0.65, P = 0.06), whereas the subgroup with low or unclear ACSM adherence demonstrated a pooled SMD = −0.15 (95% CI: −0.54, 0.25, P = 0.46). The test for subgroup differences indicated no statistically significant difference between exercise interventions with high and low ACSM adherence (P = 0.08) (Funnel plot, Forest plot, subgroup Forest plot see **Supplementary Figure 6**). These results suggest that, among older adults with sarcopenia, higher adherence to ACSM recommendations does not confer a clear advantage over lower or uncertain adherence in improving lean body mass.

##### 3.4.2.5 Body fat percentage

Heterogeneity testing revealed a high degree of heterogeneity (I²=88%, P<0.001); therefore, a random-effects model was applied. The pooled analysis showed an overall SMD =−0.62 (95% CI: −1.07,−0.17, P = 0.007), indicating that exercise interventions significantly reduced body fat percentage. In the subgroup analysis, studies were stratified according to adherence to the ACSM recommendations. The subgroup with high ACSM adherence demonstrated a pooled SMD = −0.75 (95% CI: −1.25,−0.24, P = 0.004), whereas the subgroup with low or unclear ACSM adherence yielded a pooled SMD = −0.17 (95% CI: −0.89,0.55, P = 0.65). The test for subgroup differences indicated no statistically significant difference between exercise interventions with high and low ACSM adherence (P = 0.20) (Funnel plot, Forest plot, subgroup Forest plot see **Supplementary Figure 7**). These findings suggest that, although exercise interventions are effective in reducing body fat percentage in older adults with sarcopenia, higher adherence to ACSM recommendations does not provide a clear additional benefit compared with lower or uncertain adherence.

##### 3.4.2.6 Bone mineral density

Heterogeneity testing revealed a high level of heterogeneity (I²=80%, P<0.001); therefore, a random-effects model was applied. The pooled analysis showed an overall SMD = 0.39 (95% CI:−0.25,1.03, P = 0.23), indicating that exercise interventions did not result in a statistically significant improvement in bone mineral density. In the subgroup analysis, studies were stratified according to adherence to the ACSM recommendations. The subgroup with high ACSM adherence demonstrated a pooled SMD = 0.62 (95% CI: 0.00, 1.23, P = 0.05), whereas the subgroup with low or unclear ACSM adherence showed a pooled SMD =−0.30 (95% CI: −0.69, 0.10, P = 0.14). The test for subgroup differences indicated a statistically significant difference between exercise interventions with high and low ACSM adherence (P = 0.01) (Funnel plot, Forest plot, subgroup Forest plot see **Supplementary Figure 8**). These results suggest that, among older adults with sarcopenia, exercise interventions with high adherence to ACSM recommendations are more effective in improving bone mineral density than those with lower or uncertain adherence.

#### 3.4.3 Health status and quality of life

Heterogeneity testing indicated a high degree of heterogeneity (I²=89%, P<0.001); therefore, a random-effects model was applied. The pooled analysis yielded an overall SMD = 0.45 (95% CI: -0.10,1.00, P = 0.11), indicating that exercise interventions did not result in a statistically significant improvement in health status and quality of life. In the subgroup analysis, studies were stratified according to adherence to the ACSM recommendations. The subgroup with high ACSM adherence demonstrated a pooled SMD = 0.64 (95% CI: −0.01, 1.29, P = 0.05), whereas the subgroup with low or unclear ACSM adherence showed a pooled SMD = -0.27 (95% CI: −0.73, 0.19, P = 0.25). The test for subgroup differences revealed a statistically significant difference between exercise interventions with high and low ACSM adherence (P = 0.03) (Funnel plot, Forest plot, subgroup Forest plot see **Supplementary Figure 9**). These findings suggest that, among older adults with sarcopenia, exercise interventions with high adherence to ACSM recommendations are more effective in improving health status and quality of life than those with lower or uncertain adherence.

### 3.5 Publication bias assessment

Funnel plots were constructed for handgrip strength, gait speed, walking test, skeletal muscle index, Body Mass body mass index, lean body mass, body fat percentage, bone mineral density, and health status and quality of life, and publication bias was assessed using Begg’s rank correlation test and Egger’s linear regression test. Visual inspection of the funnel plots suggested potential publication bias for health status and quality of life, whereas the remaining outcomes indicated a low likelihood of publication bias. Begg’s rank correlation test identified evidence of publication bias for the walking test (P = 0.037), while no significant bias was detected for the other outcomes (P>0.05). Egger’s linear regression test indicated publication bias for Body Mass (P = 0.037) and for health status and quality of life (P = 0.045), with no significant evidence of publication bias for the remaining outcomes (P>0.05). According to the results of publication bias tests, the Duval and Tweedie trim-and-fill method was further used to correct the outcome indicators for which Egger’s and Begg’s test suggested potential publication bias ((p<0.05)). The results of trim-and-fill analysis showed that the pooled effect size of the walking test was −0.748, with a 95% CI of −1.169 to −0.327, and the difference was statistically significant ((z=-3.484), (p<0.001)); the pooled effect size of Body Mass was −0.135, with a 95% CI of −0.506 to 0.236, and the difference was not statistically significant ((z=-0.714), (p=0.475)); the pooled effect size of health status and quality of life was 0.454, with a 95% CI of −0.096 to 1.004, and the difference was also not statistically significant ((z=1.619), (p=0.105)). Overall, no substantial changes were observed in the pooled effects and statistical conclusions of each outcome indicator after trim-and-fill analysis, indicating that potential publication bias had a limited impact on the Meta-analysis results and the findings were relatively robust are shown in **Supplementary Figures 1****–****3**.

### 3.6 Sensibility analysis

Leave-one-out sensitivity analyses were conducted for each outcome by sequentially excluding one study at a time and recalculating the pooled effect. The pooled estimates for all outcomes remained stable, with no substantial change in the magnitude, direction, or statistical significance of the standardized mean differences. These results indicate that no single study exerted a disproportionate influence on the pooled findings and that the results of this meta-analysis are robust are shown in **Supplementary Figures 4****–****13** in **Supplementary Table 1**.

### 3.7 Meta-regression analysis

This meta-analysis conducts a meta-regression analysis involving sex, age, Duration, frequency, ACSM compliance, and incomplete reporting, the meta-regression analysis revealed that the influencing factors varied across different outcome measures. Among gender composition, the proportion of females was negatively correlated with BMI (β=–0.0087, P = 0.015); the ACSM adherence rate was positively correlated with grip strength (β= 0.0151, P = 0.040) and walking speed (β= 0.0206, P = 0.042), suggesting that higher adherence to exercise prescriptions may be associated with better muscle strength and walking ability. The proportion of incomplete reporting was negatively correlated with grip strength (β=–0.0108, P = 0.033). Additionally, the ACSM adherence rate was negatively correlated with the walking test, while the proportion of incomplete reporting was positively correlated with the walking test; however, neither correlation reached statistical significance (P> 0.05). For the remaining factors including age, intervention duration, exercise frequency, and the associations between these factors and health status, quality of life, body fat percentage, lean body mass, skeletal muscle index, or bone density no statistically significant associations were found (all P> 0.05). As presented in **Supplementary Files 3**.

### 3.8 Subgroup analysis

This meta-analysis conducted subgroup analyses based on different types of physical activity; the results revealed that the impact of various exercise modalities on the various outcome measures varied to some extent. Subgroup comparisons showed statistically significant differences in health status, quality of life, BMI, grip strength, walking test performance, and walking speed (P<0.05), suggesting that the type of physical activity may exert a moderating effect on the intervention efficacy of these outcomes. Specifically, resistance training demonstrated a more pronounced improvement in grip strength and walking speed (both P<0.001) and also exerted a significant promoting effect on the walking test performance (P<0.001); combined resistance and cardiopulmonary exercise showed statistically significant effects on BMI and walking speed (P = 0.005 and P<0.001, respectively). Additionally, flexibility exercises exhibited a significant effect on health status and quality of life (P<0.001); however, due to the small sample size in some subgroups, these findings should be interpreted with caution. There were no statistically significant subgroup differences in weight, body fat percentage, lean mass, or skeletal muscle index (all P>0.05), indicating that the impact of exercise type on these outcomes has not yet shown clear variation. Overall, the pooled effect sizes for most outcomes exhibited moderate to high heterogeneity (I²=49.6%–88.7%); therefore, the results should be interpreted with caution, considering the limited number of studies, imbalanced subgroup sample sizes, and potential clinical heterogeneity as presented in **Supplementary Files 4**.

This meta-analysis conducted subgroup analyses based on different ACSM adherence rates (60%,70%,75%, and 80%). The results indicated that the high-adherence group generally demonstrated more favorable intervention outcomes compared to the low-adherence or unclear-adherence group; this trend was particularly evident across outcomes such as health status and quality of life, grip strength, walking ability, and walking speed. When the ACSM adherence rate threshold was increased from 70% to 80%, the effect size for grip strength in the high-adherence group increased from SMD = 0.59 to 0.67, and the effect size for walking speed increased from SMD = 0.85 to 0.96; the effect sizes for health status and quality of life also increased from 0.54 to 0.64. The high-adherence group exhibited statistically significant improvements across multiple outcomes including grip strength, walking speed, skeletal muscle index, and body fat percentage—whereas the effect sizes in the low-adherence or unclear-adherence groups were relatively smaller or did not reach statistical significance. The differences between subgroups reached or approached statistical significance (P = 0.01–0.08) for certain outcomes, including health status and quality of life, grip strength, walking test performance, walking speed, and bone density; these findings suggest that ACSM adherence may exert a moderating effect on the efficacy of exercise interventions. However, the subgroup differences for some outcomes were not statistically significant, and the results showed variability across different adherence threshold levels; therefore, these findings should be interpreted with caution, taking into account the number of studies, sample size, and study heterogeneity. This paper presents the results using the 75% adherence rate as the representative value; the statistical results for all other groups are presented in **Supplementary Files 5**.

## 4 Discussion

In this systematic review, a meta-analysis was conducted of 33 RCTs including a total of 2,217 older adults with sarcopenia. The findings demonstrated that exercise interventions significantly improved handgrip strength, gait speed, walking test performance, skeletal muscle index and body fat percentage in this population. Importantly, this study further evaluated adherence to ACSM-recommended exercise prescriptions and showed that, compared with low adherence, high adherence was associated with greater improvements in handgrip strength, gait speed, walking test performance, skeletal muscle index, bone mineral density and health status and quality of life. By examining exercise effects on clinical outcomes in older adults with sarcopenia based on ACSM guidelines, this study provides evidence-based insights to inform the development and optimization of exercise intervention strategies for the management of sarcopenia.

The results of this study indicate that, with respect to physical function, exercise interventions significantly improved handgrip strength, gait speed, and walking test. These findings are consistent with the meta-analysis reported by Linqian Lu et al (Lu et al., 2021). Multiple studies have demonstrated that walking or resistance training can enhance handgrip strength, increase gait speed, and shorten walking test duration in patients with sarcopenia (Seo et al., 2021; Yuenyongchaiwat and Akekawatchai, 2022; Liu et al., 2024; Rezaei et al., 2024). In terms of body composition, exercise interventions significantly improved the skeletal muscle index, while no significant effects were observed for Body Mass, body mass index, lean body mass, body fat percentage, or bone mineral density. A Cochrane meta-analysis concluded that progressive resistance training is an effective intervention for improving physical function in older adults, with benefits including increased muscle strength and muscle mass (Liu and Latham, 2009).In addition, an RCT showed that home-based exercise significantly improved the skeletal muscle index in patients with sarcopenia (Maruya et al., 2016).Regarding health status and quality of life, the overall pooled effect of exercise interventions was not statistically significant (P = 0.11). However, in the subgroup analysis, interventions with high adherence to ACSM recommendations showed a larger effect (P = 0.05) with a significant between-subgroup difference (P = 0.03), a pattern directionally consistent with the findings reported by Cristina Flor-Rufino et al (Flor-Rufino et al., 2023a).Previous studies have indicated that sarcopenia has the greatest impact on domains of physical function, such as mobility, self-care, and activities of daily living, and that these domains are particularly sensitive to high-intensity resistance training (HIRT) interventions (Go et al., 2013; Patel et al., 2013; Beaudart et al., 2015). Furthermore, evidence suggests that combining HIRT with cool-down activities based on self-massage and stretching may help alleviate pain in patients with sarcopenia (Barrachina-Igual et al., 2021).

Although the pooled standardized mean differences indicate statistically significant benefits for several outcomes, their clinical meaningfulness requires separate consideration, because a standardized effect cannot be translated directly into the units in which each outcome is measured. Gait speed is the outcome for which clinically meaningful thresholds are best established in older adults, with a change of approximately 0.05 m/s regarded as a small meaningful change and 0.10 m/s as a substantial change in community-dwelling older people (Perera et al., 2006). In the present analysis, the effect on gait speed was the largest of all outcomes (SMD = 0.70) and was greatest in the high-adherence group (SMD = 0.90), whereas the estimate in the low-adherence group was smaller and did not reach statistical significance (SMD = 0.30), suggesting that the interventions that met ACSM-recommended thresholds were of a magnitude likely to be perceptible to patients, whereas the lower-dose interventions may not have been. For handgrip strength, the pooled effect was more modest (SMD = 0.47), and reported estimates of the minimal clinically important difference for grip strength in older adults are of the order of 5.0-6.5 kg (Bohannon, 2019); because measurement protocols, dynamometer types, and participant characteristics varied considerably across the included trials, we cannot determine what proportion of the pooled effect would correspond to a change of this magnitude, and this uncertainty should be acknowledged when the results are translated into practice. More broadly, minimal clinically important differences have not been formally established for skeletal muscle index, body fat percentage, bone mineral density, or quality of life in older adults with sarcopenia, which limits the interpretation of both the statistically significant and the non-significant pooled effects reported here. Establishing such thresholds, and validating them in sarcopenic populations, should be regarded as a priority for future research, since it would allow meta-analytic estimates to be interpreted in terms of patient-relevant benefit rather than statistical significance alone.

Subgroup analyses based on the ACSM recommendations, using a 75% adherence threshold, showed that older adults with sarcopenia in the high-adherence group experienced more pronounced improvements in handgrip strength, gait speed, walking test performance, skeletal muscle index, bone mineral density, and health status and quality of life compared with those in the low-adherence group. In contrast, no significant improvements were observed for Body Mass, body mass index, lean body mass or body fat percentage. These findings indicate that strict adherence to ACSM exercise prescriptions can effectively enhance physical function, body composition, and quality of life in older adults with sarcopenia. Moreover, the subgroup analyses highlight that different exercise doses exert differential effects on rehabilitation-related outcomes in this population, underscoring the importance of referencing ACSM recommendations when developing exercise prescriptions in clinical practice to ensure more scientifically grounded and effective intervention strategies.

The multivariate meta-regression provided complementary evidence regarding the role of ACSM adherence. Higher adherence rates were independently associated with better handgrip strength (β = 0.0151, P = 0.040) and faster gait speed (β = 0.0206, P = 0.042), whereas a higher proportion of incomplete reporting of exercise dose was associated with lower handgrip strength (β= −0.0108, P = 0.033). The latter finding is of particular methodological importance, since it suggests that the association between guideline-concordant exercise and better muscle strength is attenuated when exercise dose is incompletely reported a pattern consistent with dilution of a true effect rather than with an absence of benefit. Apart from an inverse association between the proportion of female participants and body mass index, no other covariate, including age, intervention duration, and exercise frequency, was significantly associated with any outcome. Taken together, these analyses reinforce the central premise of this review: it is not only the type of exercise that matters, but also the completeness with which the exercise prescription is defined, reported, and delivered.

A review of previous meta-analyses and related studies indicates that research on older adults with sarcopenia has largely focused on specific exercise modalities, such as walking (Yuenyongchaiwat and Akekawatchai, 2022)or Baduanjin (Yang et al., 2025), or on particular types of exercise, including cardiorespiratory exercise (Najafi et al., 2018; Jung et al., 2019), resistance training (Vasconcelos et al., 2016), and whole-body vibration (Wei et al., 2017). Consequently, existing evidence allows the general conclusion that exercise interventions are beneficial for improving outcomes in older adults with sarcopenia; however, there is currently insufficient evidence to support the superiority of any single exercise program over others. In this context, the present study examined the effects of different exercise doses on rehabilitation outcomes in older adults with sarcopenia based on the ACSM exercise guidelines, thereby providing a more robust and scientifically grounded reference for the development of clinical exercise prescriptions for this population.

A key aspect of this study concerns the interpretation of adherence to ACSM recommendations. The ACSM advocates exercise prescriptions that encompass cardiorespiratory exercise, resistance training, and flexibility exercise, with detailed specifications regarding the recommended dose for each modality. However, in RCTs involving older adults with sarcopenia, descriptions of exercise dose were often incomplete or attributable only to specific intervention components. For example, two studies reported only the dose of cardiorespiratory exercise, 21 studies reported only the dose of resistance training, and only three studies provided a comprehensive classification of exercise dose that fully conformed to ACSM recommendations. In addition, several studies did not report the dose of insufficient exercise interventions. As a result, even exercise interventions that were highly consistent with ACSM recommendations may have been misclassified as low adherence or unclear adherence. Similar to pharmacological interventions, a detailed description of the exercise prescription is essential for determining an appropriate and effective dose range. Although individualization is necessary during implementation, exercise programs should be adjusted within a scientifically justified and recommended prescription framework (Bishop et al., 2025).

Several further observations support the interpretation that this gradient reflects a genuine dose response relationship rather than an artefact of the classification procedure. First, the subgroup analyses were repeated at adherence thresholds of 60%, 70%, 75%, and 80%; the high-adherence group continued to show more favorable estimates than the low- or unclear-adherence group across all four thresholds, and for handgrip strength, gait speed, and health status and quality of life the pooled effects increased monotonically as the threshold became more stringent, rising from SMD = 0.59 to 0.67, from 0.85 to 0.96, and from 0.54 to 0.64, respectively, when the threshold was raised from 70% to 80%. A monotonic gradient of this kind is difficult to attribute to random misclassification alone. Second, as described above, the meta-regression showed that the proportion of studies with incomplete reporting was inversely associated with the estimated effect on handgrip strength, implying that underreporting would have biased estimates towards the null; the true advantage of guideline-concordant exercise may therefore have been underestimated rather than overestimated. Third, the pooled estimates were stable in the leave-one-out sensitivity analyses and were essentially unchanged after trim-and-fill adjustment for the outcomes flagged for potential publication bias, indicating that the between-subgroup differences are unlikely to be driven by a small number of influential studies or by selective publication. Nevertheless, because adherence to ACSM recommendations was operationalized *post hoc* by the review team rather than defined *a priori* in the primary trials, and because only three of the 33 included studies reported exercise doses in a form that fully conformed to the ACSM recommendations, adherence should be regarded as a pragmatic proxy for exercise dose rather than a validated exposure measure, and residual misclassification of individual studies cannot be excluded.

This study has several limitations that may have contributed to potential bias in the findings. First, exercise interventions classified as having high ACSM adherence encompassed a wide range of modalities, including cycling, resistance training, flexibility training, low-intensity walking, and squats, which may have introduced substantial between-study heterogeneity. Second, considerable variability existed in intervention frequency, intensity, and duration across studies, making it difficult to directly compare interventions or to recommend a universal standard for optimal exercise prescriptions. Previous meta-analyses and RCTs have rarely compared exercise intensity and frequency, which limits the ability to identify and design optimal exercise programs for older adults with sarcopenia, including exercise type, dose, and duration. In addition, each included study carried an inherent risk of bias, and any unclear or high-risk bias domains may have inflated the estimated intervention effects. The overall risk of bias in this review was likely driven primarily by the involvement of participants and intervention providers, followed by biases related to outcome assessment and reporting. Fifth, the risk-of-bias assessment performed with the RoB 2.0 tool showed that no included trial was at low overall risk of bias: 18 studies (54.5%) were judged to be at high risk and 15 (45.5%) to raise some concerns, with deviations from the intended interventions, missing outcome data, and selection of the reported result being the domains most frequently implicated. Because exercise interventions cannot be blinded and because several trials reported outcomes only for participants who completed the intervention, the pooled effects reported here are likely to be, if anything, optimistic; the same concern applies to the between-subgroup comparisons, in which an uneven distribution of risk of bias across the adherence subgroups may have amplified the apparent advantage of high adherence. Sixth, the substantial heterogeneity observed for most outcomes (I² = 50–88%) was only partially explained by the subgroup and meta-regression analyses, indicating that unmeasured differences between trials including the diagnostic criteria used to define sarcopenia, the severity of muscle impairment at baseline, the degree of supervision and progression of training, and concurrent nutritional or pharmacological co-interventions are likely to have contributed to the residual inconsistency and may limit the generalizability of the pooled estimates. Finally, although no formal language restriction was imposed during the database searches, all included trials were published in English. Relevant trials published in other languages may therefore have been missed, and the possibility of language bias cannot be excluded; our findings should be interpreted with this caveat in mind.

These findings carry several implications for clinical practice and for future research. For clinicians, the results support prescribing exercise at doses that meet the ACSM recommended thresholds for frequency, intensity, and duration, with resistance-based and combined resistance cardiorespiratory programs representing the most consistent options for improving muscle strength and walking capacity in older adults with sarcopenia. Where resources permit, programs should be supervised and progressively adjusted, since the largest and most consistent effects were observed in trials whose exercise dose was completely reported and guideline-concordant, and adherence to the prescribed program should be monitored as a modifiable determinant of outcome rather than treated as a fixed characteristic of the patient. This approach is consistent with the physical activity recommendations for older adults issued by the World Health Organization, which likewise combine a minimum weekly volume of aerobic activity with at least two sessions of muscle-strengthening activity per week. For future trials, two priorities emerge from the present work. First, exercise dose should be described in full accordance with the FITT principle, following the reporting framework proposed in the updated ACSM position stand (Yang et al., 2025), so that adherence can be assessed prospectively rather than inferred retrospectively from incomplete descriptions. Second, trials should be designed with sufficient sample size and follow-up to determine whether the improvements in physical function and body composition observed here translate into reductions in falls, hospitalization, and mortality, and whether they persist after the intervention is withdrawn. The absence of an overall effect on health status and quality of life despite consistent effects on physical function further suggests that quality of life should be treated as a co-primary rather than a secondary outcome in future studies, and that the domains of health status most responsive to exercise in this population should be identified *a priori*.

## 5 Conclusion

This meta-analysis supports the view that exercise is an effective intervention for improving physical function and body composition in older adults with sarcopenia, and that improvements in health status and quality of life are primarily observed with high adherence to ACSM recommended exercise prescriptions, and our findings further reinforce this conclusion. When examining the optimal exercise dose for this population, we observed that exercise interventions with high adherence to ACSM recommendations were more effective than those with low adherence in improving body composition, physical function, and quality of life. These results provide valuable evidence to inform future clinical practice in the implementation of optimally dosed exercise interventions for older adults with sarcopenia.

## Data Availability

The original contributions presented in the study are included in the article/**Supplementary Material**. Further inquiries can be directed to the corresponding author.
