# Supplementary material for: Effects of exercise interventions based on American College of Sports Medicine recommendations on physical function, body composition, and quality of life in older adults with sarcopenia: a meta-analysis of randomized controlled trials
Source: Front Physiol. 2026 Oct 9;17:1882998. doi: 10.3389/fphys.2026.1882998 (PMC13690546; doi:10.3389/fphys.2026.1882998)
Supplement: Supplementary file 1 [file Supplementaryfile1.doc]

**Table 1.** Characteristics of the studies included in the meta-analysis(**supplementary files 1**)

| Author | Country | Year | Population | Age(mean+SD) | Total/male/ female | Intervention | Control | Outcome |
| --- | --- | --- | --- | --- | --- | --- | --- | --- |
| Cristina Flor-Rufino | Spain | 2023 | sarcopenia | T:79.9(7.2) C:79.6(7.7) | T:20/0/20 C:18/0/18 | High-intensity resistance training  Length of Intervention:6 months  Freq:2 times a week  Duration:65 min | CON | Health status and quality of life |
| Kornanong | Tailand | 2022 | sarcopenia | T:69.23(6.71) C:71.93(5.19) | T:28/NA/NA C:29/NA/NA | Walking-based home program  Length of Intervention:12 weeks  Freq:5 times a week  Duration:walking ≥7500 steps daily | CON | TUG; Gait speed; SMI |
| Karina S | Brazil | 2016 | sarcopenia | T:72(4.6) C:72(3.6) | T:14/NA/NA C:14/NA/NA | Progressive resistance exercise program  Length of Intervention:10 weeks  Freq:2 times a week  Duration:60 min | CON | Health status and quality of life; Gait speed |
| Won-Sang Jung | Korea | 2022 | sarcopenia | T:75.36(4.50) C:74.64(5.77) | T:14/NA/NA C:14/NA/NA | Circuit Training  Length of Intervention:12 weeks  Freq:3 times a week  Duration:45-75 min | CON | Body weight ; BMI; Body fat(%) |
| Yong‑Yao Wu | China | 2024 | sarcopenia | T:NA C:NA | T:25/19/6 C:24/17/7 | muscle relaxation training  Length of Intervention:12 weeks  Freq:3 times a week  Duration:15-20 min | CON | Health status and quality of life; HGS; sit-to-stand test; SMI |
| Sohrab Rezaei | Iran | 2024 | sarcopenia | T:72.5(4.17) C:76.5(3.53) | T:10/10/0 C:9/9/0 | High-intensity resistance training  Length of Intervention:12 weeks  Freq:3 times a week  Duration:15-20 min | CON | TUG; Body weight; BMI; chair stand test; Body fat(%) |
| Chun-De Liao | China | 2018 | sarcopenia | T:66.67(4.54) C:68.32(6.05) | T:33/0/33 C:23/0/23 | TRX suspension training  Length of Intervention:8 weeks  Freq:2 times a week  Duration:60 min | CON | Health status and quality of life; TUG; chair stand test; Body fat(%) ; SMI |
| Theresa Lichtenber | China | 2019 | sarcopenia | T:77.8(3.6) C:79.2(4.7) | T:19/19/0 C:21/21/0 | High-intensity resistance training  Length of Intervention:28 weeks  Freq:2 times a week  Duration:NA | CON | Gait speed; SMI |
| Minjing Liu | China | 2024 | sarcopenia | T:74.2(4.67) C:75.6(6.35) | T:41/12/29 C:45/18/27 | Graded Progressive Home-Based  Resistance Combined with Aerobic  Exercise  Length of Intervention:12 weeks  Freq:7 times a week  Duration:5 hours | CON | Health status and quality of life; TUG; chair stand test; Gait speed ; Body fat(%)； SMI |
| Yen-An Lin | taiwan | 2022 | sarcopenia | T:79.9(7.2) C:79.6(7.7) | T:41/NA/NA C:36/NA/NA | A group kickboxing training  Length of Intervention:12 weeks  Freq:2 times a week  Duration:90 min | CON | BMI; Gait speed ; Body fat(%) ; Total lean body mass ;  SMI; BMD |
| Wolfgang Kemmler1 | Germany | 2020 | sarcopenia | T:77.8(3.6) C:79.2(4.7) | T:19/19/0 C:21/21/0 | High-intensity resistance training  Length of Intervention:36 weeks  Freq:3 times a week  Duration:90 min | CON | Bodyfat(%) ; Total lean body mass |
| Min-Kyun Oh | Korea | 2020 | sarcopeniawith Hip Fracture | T:76.94(9.43) C:81.15(4.90) | T:19/6/13 C:19/6/13 | Antigravity Treadmill and Conventional Rehabilitation  Length of Intervention:10 days  Freq:everyday Duration: 50 min | CON | Health status and quality of life |
| Myong-Won Seo | Korea | 2021 | sarcopenia | T:70.3(5.38) C:72.9(4.7) | T:12/0/12 C:10/0/10 | High-intensity resistance training  Length of Intervention:16 weeks  Freq:3 times a week  Duration:60 min | CON | TUG; Body weight ; BMI; chair stand test; Gait speed ; Body fat(%); Total lean body mass; BMD |
| Yu-Hsuan Chien | Taiwan | 2022 | sarcopeniawith Type 2 Diabetes | T:67.6(7.7) C:67.3(6.1) | T:20/5/15 C:20/2/18 | Progressive Sandbag Exercise training  Length of Intervention:12 weeks  Freq:3 times a week  Duration:30 min | CON | Health status and quality of life ; HGS; SMI |
| Hyuma Makizako | Japan | 2020 | sarcopenia | T:74.1(6.6) C:75.8(7.3) | T:36/10/26 C:36/11/25 | Multicomponent Exercise Program  Length of Intervention:12 weeks  Freq:1 times a week  Duration:60 min | CON | sit-to-stand test; Gait speed |
| Zohre Najafi | Iran | 2018 | sarcopenia | T:NA C:NA | T:35/12/23 C:28/10/18 | Fun Physical Activities  Length of Intervention:8 weeks  Freq:3 times a week  Duration:30 min | CON | TUG |
| Elena Monti | Italy | 2023 | sarcopenia | T:78.0(6.1) C:79.6(5.8) | T:24/NA/NA C:21/NA/NA | exercise training on neuromuscular system  Length of Intervention:2 years  Freq:3 times a week  Duration:NA | CON | sit-to-stand test; Gait speed |
| Bo-yuan Chen | China | 2023 | sarcopenia | T:65.68(2.5) C:65.21(2.6) | T:25/0/25 C:24/0/24 | High-intensity resistance training  Length of Intervention:8 weeks  Freq:3 times a week  Duration:60 min | CON | sit-to-stand test ; Gait speed; SMI |
| Chun-De Liao | Taiwan | 2017 | sarcopenia | T:66.39(4.49) C:68.42(5.86) | T:25/0/25 C:21/0/21 | Elastic resistance exercise  Length of Intervention:12 weeks  Freq:3 times a week  Duration:50-55 min | CON | TUG; Gait speed ; Body fat(%) |
| Sanna Vikberg | Sweden | 2018 | sarcopenia | T:70.9(0.28) C:70.0(0.29) | T:36/16/20 C:34/18/16 | Resistance training  Length of Intervention:10 weeks  Freq:3 times a week  Duration:45 min | CON | TUG; HGS; sit-to-stand test; Total lean body mass |
| Wolfgang Kemmler2 | Germany | 2020 | sarcopenia | T:77.8(3.6) C:79.2(4.7) | T:21/21/0 C:22/22/0 | High-intensity resistance training  Length of Intervention:6 months  Freq:2 times a week  Duration:NA | CON | SMI; BMD |
| Wolfgang Kemmler3 | Germany | 2020 | sarcopenia | T:77.8(3.6) C:79.2(4.7) | T:21/21/0 C:22/22/0 | High-intensity resistance training  Length of Intervention:6 months  Freq:2 times a week  Duration:NA | CON | BMI |
| Yaqin Xiao | China | 2023 | sarcopeniawith intestinal failure | T:53.9(14.5) C:51.2(12.9) | T:30/13/17 C:30/17/13 | High-intensity resistance training  Length of Intervention:4 weeks  Freq:6 times a week  Duration:NA | CON | Health status and quality of life; Total lean body mass;  SMI; BMD |
| Hung-Ting Chen | Taiwan | 2018 | sarcopenia | T:66.7(5.3) C:68.3(2.8) | T:17/0/17 C:16/0/16 | Kettlebell training  Length of Intervention:3 months  Freq:2 times a week  Duration:60 min | CON | Body weight |
| Ekin Ilke Sen | Turkey | 2021 | sarcopenia | T:73.0(4.8) C:72.7(5.0) | T:46/NA/NA C:44/NA/NA | Home-Based Exercise Program  Length of Intervention:3 months  Freq:3 times a week  Duration:60 min | CON | Health status and quality of life; TUG |
| KOHEI MARUYA | Japan | 2016 | sarcopenia | T:69.2(5.6) C:68.5(6.2) | T:34/15/19 C:18/8/10 | High-intensity resistance training  Length of Intervention:6 months  Freq:every day  Duration:65 min | CON | Health status and quality of life; BMI; Gait speed ; Body fat(%); SMI |
| Cristina Flor-Rufino | Spain | 2022 | sarcopenia | T:79.9(7.2) C:79.6(7.7) | T:20/0/20 C:18/0/18 | High-intensity resistance training  Length of Intervention:6 months  Freq:2 times a week  Duration:NA | CON | Body weight; BMI; Gait speed ; SMI |
| Bothaina H | Australian | 2016 | sarcopenia | T:85.7(7.0) C:86.1(8.2) | T:20/NA/NA C:21/NA/NA | Resistance training  Length of Intervention:6 months  Freq:2 times a week  Duration:60 min | CON | Body weight ; BMI; Gait speed ; Body fat(%); Total lean body mass; SMI |
| Won-Sang Jung | Korea | 2019 | sarcopenia | T:75.0(3.9) C:74.9(5.2) | T:13/0/13 C:13/0/13 | High-intensity resistance training  Length of Intervention:12 weeks  Freq:3 times a week  Duration:25-75 min | CON | Body weight; BMI; Body fat(%) ; Total lean body mass |
| André Bonadias Gadelhaa | Brasilia | 2016 | sarcopenia | T:66.79(5.40) C:67.27(5.04) | T:69/0/69 C:64/0/64 | Resistance training  Length of Intervention:24 weeks  Freq:3 times a week  Duration:NA | CON | Body weight; BMI; Body fat(%) |
| Kanako Hamaguchi | Japan | 2017 | sarcopenia | T:60.4(2.7) C:60.6(2.3) | T:7/0/7 C:8/0/8 | Low-repetition and light power training  Length of Intervention:6 weeks  Freq:2 times a week  Duration:60 min | CON | SMI; BMD |
| Na Nie | China | 2023 | sarcopenia | T:66.76(5.15) C:67.28(5.13) | T:50/25/25 C:50/28/22 | Muscle Exercise  Length of Intervention:3 months  Freq:5 times a week  Duration:30 min | CON | BMI; Gait speed; Body fat(%) |
| Sheng Chen | China | 2024 | sarcopenia | T:79.86(7.54) C:80.11(7.79) | T:250/98/152 C:250/101/149 | Anti resistance exercise  Length of Intervention:6 months  Freq:2 times a week  Duration:50 min | CON | Gait speed;  SMI |

CON Randomized Controlled Tria,TUG Time-up-and-go Test,SMI Skeletal Muscle Index,BMI Body Mass Inde,HGS Handgrip strength,BMD Bone Mineral Density

Table 2 Exercise interventions evaluated according to the American College of Sports Medicines (ACSM) recommendations(**supplementary files 2**)

| Author, year | Cardiorespiratory exercise | | | | | | Resistance exercise | | | | | | | | Flexibility exercise | | | | | | | | | | | ACSM compliance | | | incomplete reporting | |
| --- | --- | --- | --- | --- | --- | --- | --- | --- | --- | --- | --- | --- | --- | --- | --- | --- | --- | --- | --- | --- | --- | --- | --- | --- | --- | --- | --- | --- | --- | --- |
|  | Frequency Days/week | | Intensity/ workload | | Duration (min) | | Frequency Days/week | | Intensity/ workload | | Repetition (min) | | Set (group) | | Frequency Days/week | | | | Intensity/ workload | | | Duration (min) | | | | Points/ Percent | | | Points/ Percent | |
| Cristina，Joaquín et al (2023) |  | |  | |  | | 2 | ☺ | 70% 1RM | ☺ | 45 | ☺ | 3 | ☺ | |  | | |  | | | |  | | | 8/8 | 100% | | 0 | 0 |
| Kornanong, Chareeporn et al (2022) | 5 | ☺ | NA | 😐 | NA | 😐 | 2 | ☺ | NA | 😐 | NA | 😐 | NA | 😐 | |  | | |  | | | |  | | | 9/14 | 64% | | 5/7 | 71 |
| Karina,João et al (2016) |  | |  | |  | | 2 | ☺ | 75% 1RM | ☺ | NA | 😐 | 2 | ☺ | |  | | |  | | | |  | | | 7/8 | 88% | | 1/4 | 25 |
| Jung,Kim et al (2022) | 3 | ☺ | HRR 60∼80% | ☺ | 20 | ☹ |  | |  | |  | |  | | | 3 | ☺ | | NA | 😐 | | | NA | 😐 | | 8/12 | 67% | | 2/6 | 33 |
| Yong‑Yao Wu , Yi‑Yi Gao et al(2024) |  | |  | |  | |  | |  | |  | |  | | | 3 | ☺ | | NA | 😐 | | | 2 | ☺ | | 5/6 | 83% | | 1/3 | 33 |
| Sohrab Rezaei, Rasoul Eslami et al (2024) |  | |  | |  | | 3 | ☺ | 60–65%  1RM | ☺ | 40 | ☺ | 3 | ☺ | |  | | |  | | | |  | | | 8/8 | 100% | | 0 | 0 |
| Chun-De Liao,Jau-Yih Tsauo et al (2018) |  | |  | |  | | 3 | ☺ | NA | 😐 | 40 | ☺ | 3 | ☺ | |  | | |  | | | |  | | | 7/8 | 88% | | 1/4 | 25 |
| Theresa,Simon et al (2019) |  | |  | |  | | 2 | ☺ | NA | 😐 | 8–15 reps | 😐 | 1-2 | ☺ | |  | | |  | | | |  | | | 6/8 | 75% | | 1/4 | 25 |
| Minjing Liu,Jiaojiao Li et al (2024) | 5 | ☺ | RPE 12-14 and HR 60-80% | ☺ | 10 | ☺ | 3 | ☺ | NA | 😐 | NA | 😐 | 3 | ☺ | |  | | |  | | | |  | | | 12/14 | 86% | | 2/7 | 29 |
| Lin, Chen et al (2022) |  | |  | |  | | NA | 😐 | NA | 😐 | 40-50 | ☺ | NA | 😐 | |  | | |  | | | |  | | | 5/8 | 63% | | 3/4 | 75 |
| 1 Wolfgang, Markus et al (2020) |  | |  | |  | | 3 | ☺ | NA | 😐 | NA | 😐 | 1-2 | ☺ | |  | | |  | | | |  | | | 6/8 | 75% | | 2/4 | 50 |
| Min-Kyun Oh,Jun-Il Yoo et al (2020) |  | |  | |  | | NA | 😐 | NA | 😐 | 20 | ☺ | NA | 😐 | |  | | |  | | | |  | | | 5/8 | 63% | | 3/4 | 75 |
| Seo,Jung et al (2021) |  | |  | |  | | 3 | ☺ | NA | 😐 | 60 | ☺ | 3-5 | ☺ | |  | | |  | | | |  | | | 7/8 | 88% | | 1/4 | 25 |
| Chien,Tsai et al (2022) |  | |  | |  | | 3 | ☺ | NA | 😐 | 30 | ☺ | 3 | ☺ | |  | | |  | | | |  | | | 7/8 | 88% | | 1/4 | 25 |
| Hyuma , Nakai et al (2020) | 1 | ☹ | NA | 😐 | 20-25 | ☺ | 1 | ☹ | NA | 😐 | 25-30 | ☺ | NA | 😐 | | 1 | | ☹ | NA | | 😐 | | 5 | | ☹ | 8/20 | 40% | | 4/10 | 40 |
| Zohre,Kooshiar et al (2018) | 3 | ☺ | NA | 😐 | 20 | ☺ |  | |  | |  | |  | |  | | | |  | | |  | | | | 5/6 | | 83% | 1/3 | 33 |
| Elena,Sara et al (2023) | 3 | ☺ | NA | 😐 | NA | 😐 | 3 | ☺ | NA | 😐 | NA | 😐 | NA | 😐 | | 3 | | 2 | NA | | 😐 | | NA | | 😐 | 13/20 | 65% | | 7/10 | 70 |
| Bo-yuan , Yuan-zhe et al (2023) | 3 | ☺ | NA | 😐 | NA | 😐 | 3 | ☺ | 60–80%1RM | ☺ | NA | 😐 | 2-3 | ☺ | |  | | |  | | | |  | | | 11/14 | 79% | | 3/7 | 43 |
| Chun-De,Jau-Yih et al (2017) |  | |  | |  | | 3 | ☺ | NA | 😐 | 35-40 | ☺ | 3 | ☺ | |  | | |  | | | |  | | | 7/8 | 88% | | 1/4 | 25 |
| Sanna ,Niklas et al (2018) |  | |  | |  | | 3 | ☺ | NA | 😐 | 45 | ☺ | 2-4 | ☺ | |  | | |  | | | |  | | | 7/8 | 88% | | 1/4 | 25 |
| 2 Wolfgang, Markus et al (2020 |  | |  | |  | | 3 | ☺ | NA | 😐 | NA | 😐 | 1-2 | ☺ | |  | | |  | | | |  | | | 6/8 | 75% | | 2/5 | 50 |
| 3 Wolfgang, Markus et al (2020 |  | |  | |  | | 3 | ☺ | NA | 😐 | NA | 😐 | 1-2 | ☺ | |  | | |  | | | |  | | | 6/8 | 75% | | 2/5 | 50 |
| Yaqin Xiao,Deshuai Song et al (2023) |  | |  | |  | | 6 | ☺ | NA | 😐 | NA | 😐 | 3 | ☺ | |  | | |  | | | |  | | | 6/8 | 75% | | 2/4 | 50 |
| Chen,Wu et al (2018) |  | |  | |  | | 2 | ☺ | 60%–70% 1RM | ☺ | 60 | ☺ | 3 | ☺ | |  | | |  | | | |  | | | 8/8 | 100% | | 0 | 0 |
| Ekin Ilke Sen,Sibel Eyigo et al ( 2021) | 3 | ☺ | NA | 😐 | 30 | ☺ | 3 | ☺ | NA | 😐 | 20 | ☺ | 1-2 | ☺ | | 3 | | ☺ | NA | | 😐 | | 10 | | ☺ | 17/20 | 85% | | 3/7 | 43 |
| KOHEI,YASUYOSHIet al (2016) | 7 | ☺ | NA | 😐 | 20-30 | ☺ | 7 | ☺ | NA | 😐 | NA | 😐 | 3 | ☺ | |  | | |  | | | |  | | | 10/14 | 71% | | 3/7 | 43 |
| Cristina,Joaquín et al (2022) |  | |  | |  | | 2 | ☺ | 70% 1RM | ☺ | 45 | ☺ | 3 | ☺ | |  | | |  | | | |  | | | 8/8 | 100% | | 0 | 0 |
| Bothaina,Jennifer et al (2016) |  | |  | |  | | 2 | ☺ | NA | 😐 | 20-30 | ☺ | 2-3 | ☺ | |  | | |  | | | |  | | | 7/8 | 88% | | 1/4 | 25 |
| Jung,Kim et al (2019) | 3 | ☺ | 60% -80%HRR | ☺ | 25-70 | ☺ |  | |  | |  | |  | |  | | | |  | | |  | | | | 6/6 | | 100% | 0 | 0 |
| André ,Flávio et al (2016) |  | |  | |  | | 3 | ☺ | 60–80% 1RM | ☺ | NA | 😐 | 3 | ☺ | |  | | |  | | | |  | | | 7/8 | 88% | | 1/4 | 25 |
| Hamaguchi, Kurihara et al (2017) |  | |  | |  | | 2 | ☺ | NA | 😐 | NA | 😐 | 8 | ☺ | |  | | |  | | | |  | | | 6/8 | 75% | | 2/4 | 50 |
| NaNie,Zhihua Yin et al (2023) | 5 | ☺ | NA | 😐 | 2 | 😐 | 5 | ☺ | 30 | ☺ | NA | 😐 | NA | 😐 | |  | | |  | | | |  | | | 11/14 | 79% | | 3/7 | 43 |
| Sheng Chen,Hao Zhang et al (2024) |  | |  | |  | | 2 | ☺ | NA | 😐 | 50 | ☺ | 2 | ☺ | |  | | |  | | | |  | | | 7/8 | 88% | | 1/4 | 25 |

Table 3: Meta-regression analysis of factors influencing the effectiveness of physical activity interventions(**supplementary files 3**)

| Item | Coefficient | Std.err. | t | P>\|t | [95% cl] |
| --- | --- | --- | --- | --- | --- |
| Health Status and Quality of Life | | | | | |
| sex（female/total,%) | -0.0225346 | 0.0203696 | -1.11 | 0.311 | [-0.0723771,0.0273079] |
| age (years) | 0.0126341 | 0.0170858 | 0.74 | 0.484 | [-0.0277673,0.124543] |
| Duration（week） | -0.0020767 | 0.0549078 | -0.04 | 0.971 | [-0.1286965,0.214934] |
| frequency(days/week) | -0.2035147 | 0.1814605 | -1.12 | 0.295 | [-0.6219634,0.1211374] |
| ACSM compliance(%) | 0.0259506 | 0.0412778 | 0.63 | 0.547 | [-0.0692363,0.1211374] |
| incomplete reporting（%） | -0.0160068 | 0.0216101 | -0.74 | 0.480 | [-0.0658397,0.0338262] |
| **Body Mass Index** | | | | | |
| sex（female/total,%) | -0.0087271 | 0.0025674 | -3.4 | 0.015 | [-0.0150094,-0.0024448] |
| age (years) | 0.0021895 | 0.0170109 | 0.13 | 0.9 | [-0.0362919,0.0406709] |
| Duration（week） | -0.0073046 | 0.0204572 | -0.36 | 0.729 | [-0.0535819,0.0137271] |
| frequency(days/week) | 0.1165326 | 0.091061 | 1.28 | 0.233 | [-0.0894616,0.3225268] |
| ACSM compliance(%) | -0.0115322 | 0.011166 | -1.03 | 0.329 | [-0.0367914,-0.0137271] |
| incomplete reporting（%） | 0.0038207 | 0.0063553 | 0.60 | 0.563 | [-0.010556,0.0181974] |
| **Handgrip Strength** | | | | | |
| sex（female/total,%) | -0.0065939 | 0.0053938 | -1.22 | 0.242 | [-0.0181624,0.0049745] |
| age (years) | 0.0063083 | 0.0151158 | 0.42 | 0.682 | [-0.0255833,0.0381998] |
| Duration（week） | -0.000039 | 0.0055717 | -0.01 | 0.994 | [-0.0117447,0.0116668] |
| frequency(days/week) | -0.0748749 | 0.0566634 | -1.32 | 0.203 | [-0.1939204,0.0441706] |
| ACSM compliance(%) | 0.0151222 | 0.0068359 | 2.21 | 0.040 | [0.0007605,0.0294839] |
| incomplete reporting（%） | -0.0108077 | 0.0046828 | -2.31 | 0.033 | [-0.0206459,-0.0009694] |
| **Walking Test** | | | | | |
| sex（female/total,%) | -0.0008363 | 0.0100861 | -0.08 | 0.973 | [-0.0267634,0.0250908] |
| age (years) | 0.0065826 | 0.0824648 | 0.08 | 0.939 | [-0.1952015,0.2083668] |
| Duration（week） | -0.1536309 | 0.0953539 | -1.61 | 0.151 | [-0.3791071,0.0718454] |
| frequency(days/week) | 0.0401058 | 0.1834152 | 0.22 | 0.833 | [-0.3791071,0.0718454] |
| ACSM compliance(%) | -0.0490261 | 0.0221893 | -2.21 | 0.063 | [-0.1014955,0.0034434] |
| incomplete reporting（%） | 0.0240698 | 0.0113088 | 2.13 | 0.071 | [-0.0026712,0.0508108] |
| **Body Mass** | | | | | |
| sex（female/total,%) | -0.0148908 | 0.005905 | -2.52 | 0.065 | [-0.0312858,0.0015042] |
| age (years) | 0.0181515 | 0.0245454 | 0.74 | 0.488 | [-0.0419089,0.0782119] |
| Duration（week） | -0.0251132 | 0.0323905 | -0.78 | 0.468 | [-0.1043699,0.541435] |
| frequency(days/week) | -0.0337397 | 0.4345913 | -0.08 | 0.941 | [-1.097146,1.029667] |
| ACSM compliance(%) | 0.0136883 | 0.0195698 | 0.7 | 0.51 | [-0.0341973,0.0615738] |
| incomplete reporting（%） | -0.008755 | 0.0152341 | -0.57 | 0.586 | [-0.0460314,0.0285215 |
| **Gait Speed** | | | | | |
| sex（female/total,%) | -0.0013218 | 0.0059303 | -0.22 | 0.828 | [-0.0145354,0.0118918] |
| age (years) | -0.0193312 | 0.0220866 | -0.88 | 0.395 | [-0.0664076,0.0277452] |
| Duration（week） | -0.0087396 | 0.0069746 | -1.25 | 0.229 | [-0.0236057,0.0061265] |
| frequency(days/week) | 0.0377319 | 0.0939346 | 0.4 | 0.694 | [-0.162485,0.2379488] |
| ACSM compliance(%) | 0.0206411 | 0.0092773 | 2.22 | 0.042 | [0.0008669,0.0404152] |
| incomplete reporting（%） | -0.0122631 | 0.0080334 | -1.53 | 0.148 | [-0.0293858,0.0048597] |
| Body Fat Percentage | | | | | |
| sex（female/total,%) | 0.0075587 | 0.0060602 | 1.25 | 0.248 | [-0.0064161,0.0215335] |
| age (years) | 0.0274583 | 0.0325691 | 0.84 | 0.417 | [-0.0442259,0.0991425] |
| Duration（week） | -0.0007913 | 0.0291874 | -0.03 | 0.979 | [-0.0650324,0.0634499] |
| frequency(days/week) | 0.0319633 | 0.1372274 | 0.23 | 0.82 | [-0.270072,0.3339987] |
| ACSM compliance(%) | -0.0038279 | 0.021173 | -0.18 | 0.86 | [-0.0504293,0.0427735] |
| incomplete reporting（%） | 0.0028123 | 0.0121983 | 0.23 | 0.822 | [-0.0240359,0.0296605] |
| Lean Body Mass | | | | | |
| sex（female/total,%) | -0.0085731 | 0.0051252 | -1.67 | 0.193 | [-0.024884,0.0077377] |
| age (years) | -0.0042598 | 0.0169408 | -0.25 | 0.811 | [-0.0478076,0.039288] |
| Duration（week） | 0.0237415 | 0.0146597 | 1.62 | 0.166 | [-0.0139425,0.0614255] |
| frequency(days/week) | 0.0924287 | 0.1231949 | 0.75 | 0.487 | [-0.2242539,0.4091113] |
| ACSM compliance(%) | -0.0001997 | 0.0153251 | -0.01 | 0.99 | [-0.0395942,0.0391949] |
| incomplete reporting（%） | -0.0001689 | 0.0076362 | -0.02 | 0.983 | [-0.0197983,0.0194605] |
| **Skeletal Muscle Index** | | | | | |
| sex（female/total,%) | -0.0129659 | 0.0061534 | -2.11 | 0.061 | [-0.0266766,0.0007447] |
| age (years) | 0.0008756 | 0.0251635 | 0.03 | 0.973 | [-0.0539509,.0557021] |
| Duration（week） | -0.0180044 | 0.0249989 | -0.72 | 0.484 | [-0.0720112,0.0360023] |
| frequency(days/week) | -0.0669018 | 0.1169587 | -0.57 | 0.577 | [-0.3195757,0.185772] |
| ACSM compliance(%) | -0.0022036 | 0.0217736 | -0.1 | 0.921 | [-0.0492426,0.0448355] |
| incomplete reporting（%） | 0.0020389 | 0.0109611 | 0.19 | 0.855 | [-0.021641,0.0257189] |
| Bone Mineral Density | | | | | |
| sex（female/total,%) | -0.0110624 | 0.0056197 | -1.97 | 0.188 | [-0.0352418,0.013117] |
| age (years) | 0.0092282 | 0.0336614 | 0.27 | 0.802 | [-0.0978974,0.1163539] |
| Duration（week） | -0.0297783 | 0.0786098 | -0.38 | 0.73 | [-0.2799496,.2203931] |
| frequency(days/week) | 0.0198332 | 0.2280897 | 0.09 | 0.936 | [-0.7060501,0.7457166] |
| ACSM compliance(%) | 0.0388801 | 0.0385174 | 1.01 | 0.387 | [-0.0836993,0.1614596] |
| incomplete reporting（%） | -0.0198408 | 0.0190315 | -1.04 | 0.374 | [-0.0804074,0.0407258] |

Table 4: Subgroup analysis of the efficacy of different exercise interventions(**supplementary files 4**)

| **Item** | **statistic** | **freedom** | **P** | **I-squared** | **Tau-squared** | **Z** | **P** | **sub-groups** |
| --- | --- | --- | --- | --- | --- | --- | --- | --- |
| Health Status and Quality of Life | | | | | | | | |
| Resistance exercise | 13.11 | 5 | 0.022 | 61.90% | 0.161 | 1.440 | 0.150 |  |
| Resistance exercise +Cardiorespiratory exercise +Flexibility exercise | 0 | 0 |  |  | 0.000 | 1.900 | 0.058 |  |
| Resistance exercise +Cardiorespiratory exercise | 0.74 | 1 | 0.391 | 0.00% | 0.000 | 0.040 | 0.971 |  |
| Flexibility exercise | 0 | 0 |  |  | 0.000 | 7.850 | 0.000 |  |
| Overall | 79.38 | 9 | 0 | 88.70% | 0.682 | 1.620 | 0.105 | 0.000 |
| **Body Mass Index** | | | | | | | | |
| Resistance exercise | 13.02 | 6 | 0.043 | 53.90% | 0.092 | 0.770 | 0.444 |  |
| Resistance exercise +Cardiorespiratory exercise | 0.64 | 1 | 0.423 | 0.00% | 0.000 | 2.810 | 0.005 |  |
| Cardiorespiratory exercise | 0 | 0 |  |  | 0.000 | 0.820 | 0.598 |  |
| Cardiorespiratory exercise +Flexibility exercise | 0 | 0 |  |  | 0.000 | 1.370 | 0.001 |  |
| Overall | 28.94 | 10 | 0.010 | 65.50% | 0.153 | 0.270 | 0.000 | 0.010 |
| **Handgrip Strength** | | | | | | | | |
| Resistance exercise | 34.23 | 11 | 0.000 | 67.90% | 0.142 | 3.6300 | 0.0000 |  |
| Resistanceexercise +Cardiorespiratory exercise | 7.30 | 4 | 0.121 | 45.20% | 0.054 | 2.800 | 0.005 |  |
| Resistance exercise +Cardiorespiratory exercise +Flexibility exercise | 0.45 | 1 | 0.502 | 0.00% | 0.000 | 0.530 | 0.598 |  |
| Flexibility exercise | 0.00 | 0 |  |  | 0.000 | 3.220 | 0.001 |  |
| Overall | 50.56 | 19 | 0.000 | 62.40% | 0.105 | 4.780 | 0.000 | 0.036 |
| **Walking Test** | | | | | | | | |
| Resistance exercise | 7.42 | 4 | 0.115 | 46.10% | 0.104 | 5.270 | 0.000 |  |
| Resistance exercise +Cardiorespiratory exercise +Flexibility exercise | 0.00 | 0 |  |  | 0.000 | 2.740 | 0.006 |  |
| Resistance exercise +Cardiorespiratory exercise | 4.57 | 1 | 0.033 | 78.10% | 0.208 | 1.080 | 0.279 |  |
| Cardiorespiratory exercise | 0.00 | 0 |  |  | 0.000 | 1.640 | 0.101 |  |
| Overall | 37.44 | 8 | 0.000 | 78.60% | 0.293 | 3.470 | 0.001 | 0.000 |
| **Body Mass** | | | | | | | | |
| Resistance exercise | 16.80 | 5 | 0.005 | 70.20% | 0.237 | 0.020 | 0.982 |  |
| Cardiorespiratory exercise +Flexibility exercise | 0.00 | 0 |  |  | 0.000 | 1.230 | 0.220 |  |
| Cardiorespiratory exercise | 0.00 | 0 |  |  | 0.000 | 1.270 | 0.206 |  |
| Overall | 17.46 | 7 | 0.015 | 59.90% | 0.161 | 0.710 | 0.475 | 0.720 |
| **Gait Speed** | | | | | | | | |
| Resistance exercise | 41.15 | 9 | 0.000 | 0.78% | 0.247 | 4.520 | 0.000 |  |
| Resistance exercise +Cardiorespiratory exercise | 10.71 | 4 | 0.030 | 62.60% | 0.114 | 3.820 | 0.000 |  |
| Resistance exercise +Cardiorespiratory exercise +Flexibility exercise | 0.41 | 1 | 0.521 | 0.00% | 0.000 | 0.060 | 0.956 |  |
| Overall | 65.38 | 16 | 0.000 | 75.50% | 0.194 | 5.380 | 0.000 | 0.001 |
| Body Fat Percentage | | | | | | | | |
| Resistance exercise | 26.62 | 7 | 0.000 | 80.90% | 0.342 | 2.060 | 0.040 |  |
| Resistance exercise +Cardiorespiratory exercise | 57.77 | 2 | 0.000 | 96.50% | 1.770 | 0.870 | 0.386 |  |
| Cardiorespiratory exercise | 0.00 | 0 |  |  | 0.000 | 2.530 | 0.012 |  |
| Cardiorespiratory exercise +Flexibility exercise | 0.00 | 0 |  |  | 0.000 | 2.790 | 0.005 |  |
| Overall | 97.86 | 12 | 0.000 | 87.70% | 0.580 | 2.680 | 0.007 | 0.324 |
| Lean Body Mass | | | | | | | | |
| Resistance exercise | 11.88 | 5 | 0.036 | 57.90% | 0.106 | 1.310 | 0.190 |  |
| Cardiorespiratory exercise | 0.00 | 0 |  |  | 0.000 | 0.580 | 0.560 |  |
| Overall | 11.90 | 6 | 0.064 | 49.60% | 0.082 | 1.450 | 0.148 | 0.878 |
| **Skeletal Muscle Index** | | | | | | | | |
| Resistance exercise | 63.46 | 9 | 0.000 | 85.80% | 0.377 | 2.590 | 0.009 |  |
| Resistance exercise +Cardiorespiratory exercise | 4.67 | 3 | 0.198 | 35.80% | 0.041 | 2.350 | 0.019 |  |
| Flexibility exercise | 0.00 | 0 |  |  | 0.000 | 0.650 | 0.515 |  |
| Overall | 68.63 | 14 | 0.000 | 79.60% | 0.241 | 3.230 | 0.001 | 0.781 |
| Bone Mineral Density | | | | | | | | |
| Resistance exercise | 20.47 | 4 | 0.000 | 80.50% | 0.410 | 1.190 | 0.234 |  |
| Overall | 20.47 | 4 | 0.000 | 80.50% | 0.410 | 1.190 | 0.234 | 0.000 |

Table 5: Subgroup analysis of the effectiveness of exercise interventions under different ACSM exercise prescription adherence levels**(supplementary files 5**)

| **ACSMcompliance(%)** | high ACSM adherence | low or unclear ACSM adherence | **subgroup** |
| --- | --- | --- | --- |
| **Health Status and Quality of Life** | | |  |
| 60 | None |  |  |
| 70 | SMD =0.54 (95% CI:- 0.06, 1.14, P=0.08) | SMD =-0.29 (95% CI: -0.93, 0.35, P=0.37) | p=0.06 |
| 80 | SMD =0.64 (95% CI:- 0.01, 1.29, P=0.05) | SMD =-0.27 (95% CI: -0.73, 0.19, P=0.25) | p=0.02 |
| **Body Mass Index** | | | |
| 60 | None |  |  |
| 70 | SMD =0.02 (95% CI: -0.34, 0.38, P=0.92) | SMD =0.13 (95% CI: -0.46, 0.72, P=0.66) | p=0.75 |
| 80 | SMD =-0.20 (95% CI: -0.58, 0.17, P=0.29) | SMD =0.26 (95% CI: -0.03, 0.55, P=0.08) | p=0.06 |
| **Handgrip Strength** | | | |
| 60 | SMD =0.50 (95% CI: 0.30, 0.69, P<0.0001) | SMD =0.00 (95% CI: -0.46, 0.46, P=1.0) | p=0.05 |
| 70 | SMD =0.59 (95% CI: 0.36, 0.81, P<0.001) | SMD =0.17 (95% CI: -0.07, 0.41, P=0.15) | p=0.01 |
| 80 | SMD =0.67(95% CI: 0.34, 1.00, P<0.0001) | SMD =0.34 (95% CI: 0.13, 0.55, P=0.001) | p=0.10 |
| **Walking Test** | | | |
| 60 | None |  |  |
| 70 | SMD =−0.82 (95% CI: −1.26 ,−0.39, P<0.001) | SMD =−0.02 (95% CI: −0.52 , 0.49, P<0.001) | p=0.02 |
| 80 | SMD =−0.82 (95% CI: −1.26 ,−0.39, P<0.001) | SMD =−0.02 (95% CI: −0.52 , 0.49, P<0.001) | p=0.02 |
| **Body Mass** | | | |
| 60 | None |  |  |
| 70 | SMD =−0.08 (95% CI: −0.50 , 0.34, P=0.71) | SMD = −0.49 (95% CI: −1.24 , 0.27, P=0.21) | p=0.35 |
| 80 | SMD =−0.08 (95% CI: −0.50 , 0.34, P=0.71) | SMD = −0.49 (95% CI: −1.24 , 0.27, P=0.21) | p=0.35 |
| **Gait Speed** | | | |
| 60 | SMD =0.76 (95% CI: 0.50, 1.01, P<0.0001) | SMD =-0.10 (95% CI: -0.57, 0.36, P=0.66) | p=0.001 |
| 70 | SMD =0.85 (95% CI: 0.53, 1.16, P<0.0001) | SMD =0.31 (95% CI: -0.07, 0.70, P=0.11) | p=0.04 |
| 80 | SMD =0.96(95% CI: 0.54, 1.37, P<0.0001) | SMD =0.47 (95% CI: 0.07, 0.86, P=0.02) | p=0.10 |
| **Body Fat Percentage** | | | |
| 60 | None |  |  |
| 70 | SMD =-0.64 (95% CI: -1.14, -0.14, P=0.01) | SMD =-0.49 (95% CI: -1.68, 0.70, P=0.42) | p=0.82 |
| 80 | SMD =-0.57(95% CI: -0.97, -0.17, P=0.005) | SMD =-0.74 (95% CI: -2.00, 0.52, P=0.25) | p=0.80 |
| **Lean Body Mass** | | | |
| 60 | None |  |  |
| 70 | SMD =0.32 (95% CI: −0.01 , 0.65, P=0.06) | SMD =−0.15 (95% CI: −0.54 , 0.25, P=0.46) | p=0.08 |
| 80 | SMD =0.32 (95% CI: −0.01 , 0.65, P=0.06) | SMD =−0.15 (95% CI: −0.54 , 0.25, P=0.46) | p=0.08 |
| **Skeletal Muscle Index** | | | |
| 60 | None |  |  |
| 70 | SMD =0.57 (95% CI: 0.24, 0.89, P=0.0006) | SMD =-0.02 (95% CI: -0.52, 0.49, P=0.95) | p=0.06 |
| 80 | SMD =0.30(95% CI: 0.13, 0.48, P=0.0008) | SMD =0.70 (95% CI: 0.07, 1.33, P=0.03) | p=0.23 |
| **Bone Mineral Density** | | |  |
| 60 | None |  |  |
| 70 | SMD = 0.62 (95% CI: 0.00, 1.23, P=0.05) | SMD =−0.30 (95% CI: −0.69 , 0.10, P=0.14) | p=0.01 |
| 80 | SMD =0.60(95% CI: -0.26, 1.47, P=0.17) | SMD =0.34 (95% CI: -0.42, 1.11, P=0.38) | p=0.66 |

Figure 1 Filled funnel plot with pseudo 95% confidence limits-walking test

Figure 2 Filled funnel plot with pseudo 95% confidence limits-Body Mass

Figure 3 Filled funnel plot with pseudo 95% confidence limits-walking test

health status and quality of life

Figure 4: Sensitivity Analysis Forest Plot- Health Status and Quality of Life

Figure 5: Sensitivity Analysis Forest Plot- Body Mass Index

Figure 6: Sensitivity Analysis Forest Plot- Handgrip Strength

Figure 7: Sensitivity Analysis Forest Plot- Walking Test

Figure 8: Sensitivity Analysis Forest Plot- Body Mass

Figure 9: Sensitivity Analysis Forest Plot- Gait Speed

Figure 10: Sensitivity Analysis Forest Plot- Body Fat Percentage

Figure 11: Sensitivity Analysis Forest Plot- Lean Body Mas

Figure 12: Sensitivity Analysis Forest Plot-Skeletal Muscle Index

Figure 13: Sensitivity Analysis Forest Plot-Bone Mineral Density
